# SRF deletion results in earlier disease onset in a mouse model of amyotrophic lateral sclerosis

**DOI:** 10.1172/jci.insight.167694

**Published:** 2023-08-08

**Authors:** Jialei Song, Natalie Dikwella, Daniela Sinske, Francesco Roselli, Bernd Knöll

**Affiliations:** 1 Institute of Neurobiochemistry and; 2 Department of Neurology, Ulm University, Ulm, Germany.; 3 German Center for Neurodegenerative Diseases-Ulm (DZNE-Ulm), Ulm, Germany.

**Keywords:** Neuroscience, ALS, Autophagy, Mouse models

## Abstract

Changes in neuronal activity modulate the vulnerability of motoneurons (MNs) in neurodegenerative diseases, including amyotrophic lateral sclerosis (ALS). So far, the molecular basis of neuronal activity’s impact in ALS is poorly understood. Herein, we investigated the impact of deleting the neuronal activity–stimulated transcription factor (TF) serum response factor (SRF) in MNs of SOD1^G93A^ mice. SRF was present in vulnerable MMP9^+^ MNs. Ablation of SRF in MNs induced an earlier disease onset starting around 7–8 weeks after birth, as revealed by enhanced weight loss and decreased motor ability. This earlier disease onset in SRF-depleted MNs was accompanied by a mild elevation of neuroinflammation and neuromuscular synapse degeneration, whereas overall MN numbers and mortality were unaffected. In SRF-deficient mice, MNs showed impaired induction of autophagy-encoding genes, suggesting a potentially new SRF function in transcriptional regulation of autophagy. Complementary, constitutively active SRF-VP16 enhanced autophagy-encoding gene transcription and autophagy progression in cells. Furthermore, SRF-VP16 decreased ALS-associated aggregate induction. Chemogenetic modulation of neuronal activity uncovered SRF as having important TF-mediating activity–dependent effects, which might be beneficial to reduce ALS disease burden. Thus, our data identify SRF as a gene regulator connecting neuronal activity with the cellular autophagy program initiated in degenerating MNs.

## Graphical abstract

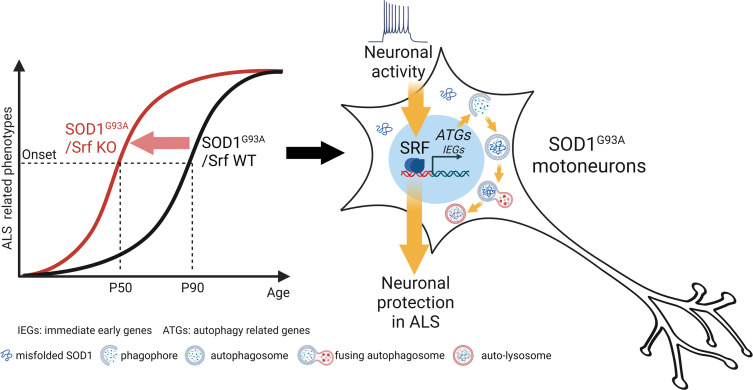

## Introduction

Motoneurons (MNs) display differences in vulnerability to the pathogenic process determining Amyotrophic Lateral Sclerosis (ALS). While fast-fatigable MNs (FF-MN) and large fatigue-resistant MNs (FR-MN) are highly affected and are the first to degenerate, small FR-MN and slow MN (S-MN) are resistant and degenerate only in part and only at the end stage (1–4). Considering that FF-MN and S-MN differ in their basic firing properties (5), as well as in their transcriptional profile (6, 7), it has been hypothesized that differences in excitability and MN activity may account for the differential vulnerability (8). Furthermore, only vulnerable FF-MNs and large FR-MNs express matrix metalloproteinase-9 (MMP9) as a marker (2). Whether MN hyper- or hypoexcitability is associated with vulnerability is currently under debate. Early observations indicate that MNs are hyperexcitable in ALS, which contributes to their slow excitotoxic death (9–12). More recent work has shown that vulnerable, FF-MNs display hypoexcitability before denervation (13–15), whereas S-MNs remain hyperexcitable (3). Similar changes in excitability have been observed in induced pluripotent stem cell–derived (iPSC-derived) MNs with different mutations (16, 17). Here, MNs show early signs of increased excitability but a decrease in excitability over time. Interestingly, restoration of excitation through chemogenetics appears to have a beneficial effect on multiple ALS-associated cellular phenotypes, including autophagy impairment, protein aggregation, ER stress, and neuromuscular junction (NMJ) denervation (17–20). In addition to these modifications of intrinsic excitability (13), disruption of synaptic inputs has been also reported in MNs (19, 20). These observations suggest that, during disease progression, vulnerable MNs shift from a stage of increased excitability to one of decreased excitability, contributing to the loss of this MN subpopulation. However, the molecular pathways linking MN excitation to their vulnerability are not well described (20).

Synaptic transmission induces activity-dependent gene expression involved in the remodeling of neuronal responses in development, memory, learning, and repair (21, 22). The serum response factor (SRF) is one of the key transcription factors (TFs) of neuronal activity–dependent gene expression (23, 24). In conjunction with coregulators of the TCF (e.g., Elk1) and MRTF (e.g., MRTF-A) family, SRF is responsible for the induction of immediate early genes (IEG) such as Fos (*c-Fos*, *Fosb*) and Egr (*Egr1*, *Egr2*, *Egr3*) family members *Arc* and *Npas4* (25, 26) during periods of increased synaptic activation. Neuronal activity targeting SRF can be elicited by ocular dominance plasticity (27), novel environments (28), drug application (29), acute stress (30, 31), and epileptic seizures (25, 26). For instance, IEG induction by epileptic seizures is diminished in brain-specific *Srf* mutant mice (25, 26).

SRF conveys neuroprotection in several neurodegenerative paradigms. In facial MNs, constitutively active SRF-VP16 protects from degeneration after injury (32) and enhances nerve regeneration (33). Similarly, SRF mediates neuroprotection against oxidative stress (34), DNA damage (35), and traumatic brain injury (36). Thus, the transcriptional programs driven by SRF appear to increase neuronal resilience to insults (32, 35). This suggests that SRF is a promising candidate that simultaneously links intrinsic neuronal excitability and synaptic inputs as well as the dysfunction of cellular processes and MN degeneration observed in ALS. However, the role of SRF in ALS has not yet been analyzed.

In order to analyze a potential neuroprotective SRF function in ALS, we engineered a conditional *Srf* mouse mutant in the context of the SOD1^G93A^ ALS model where SRF was deleted in MNs. We have demonstrated that SRF removal from MNs determines an earlier disease appearance and an accelerated disease course with no difference in overall survival. We provide a potentially new link for SRF as a gene regulator connecting neuronal activity–mediated gene expression with autophagy induction in MNs with an ALS pathology.

## Results

### SRF is present in vulnerable mouse and human MNs.

**Figure 1:**
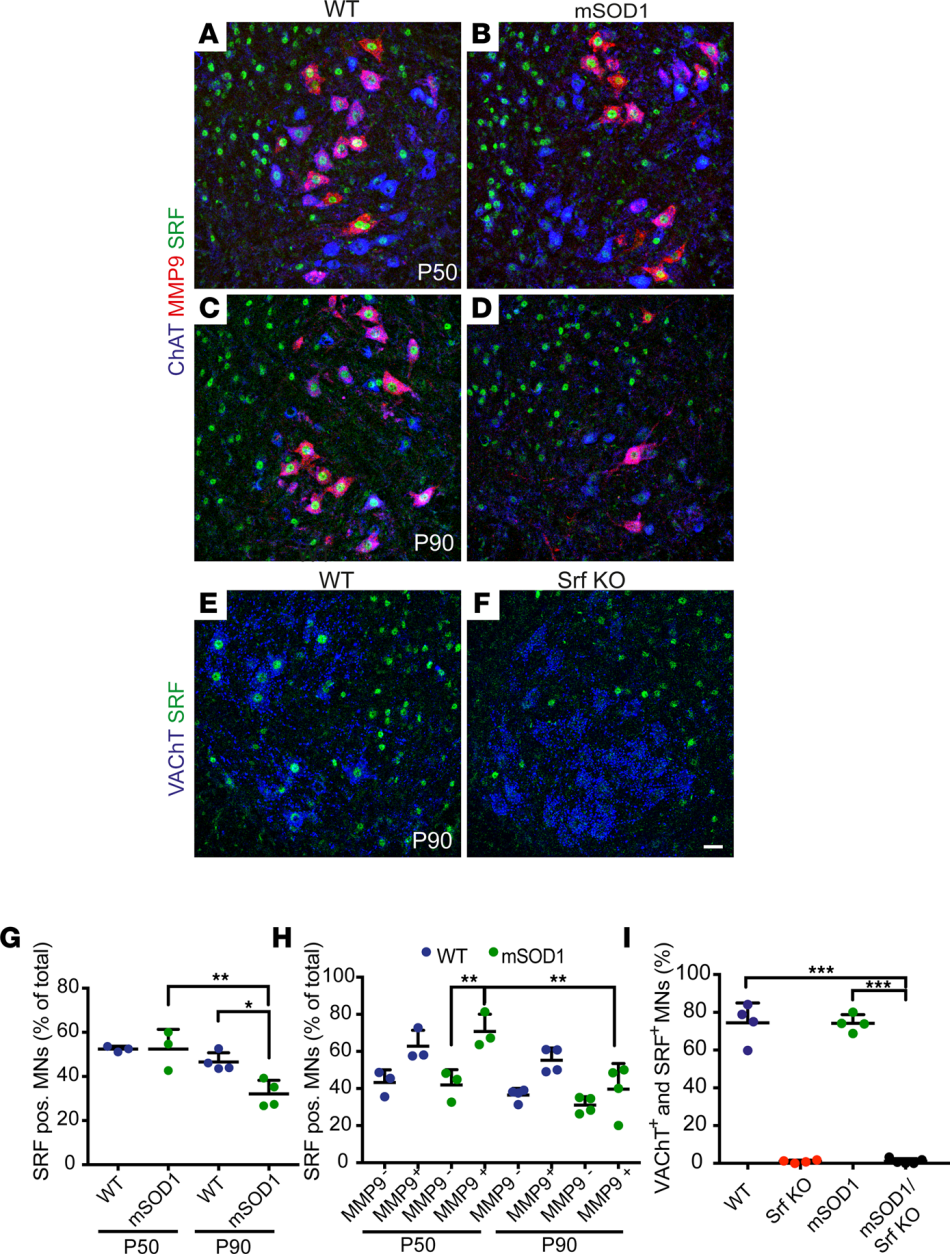
SRF is present in vulnerable MNs. (**A**–**D**) Ventral horns from P50 (**A** and **B**) or P90 (**C** and **D**) WT (**A** and **C**) or mSOD1 (**B** and **D**) mice were stained for ChAT (blue), MMP9 (red), and SRF (green). SRF was present in nonvulnerable MMP9^–^ and vulnerable MMP9^+^ MNs in WT and mSOD1 mice. (**E** and **F**) In Srf KO MNs (**F**), SRF was absent from VAChT^+^ MNs at P90 in contrast to WT MNs (**E**). (**G**) In WT and mSOD1 mice, approximately 50% of MNs were SRF^+^ at P50. At P90, abundance of SRF^+^ MNs was decreased in mSOD1 mice. There was no significant change in WT MNs between P50 and P90. (**H**) In WT mice, SRF was more present in MMP9^+^ MNs (~60%) compared with MMP9^–^ MNs (~40%). In mSOD1 mice, SRF was significantly more present in vulnerable MMP9^+^ MNs compared with MMP9^–^ MNs at P50, in contrast to P90. (**I**) SRF was nearly absent from MNs in Srf-KO and SOD1/Srf-KO mice compared with WT and SOD1 animals at P90. In **G**–**I**, each dot represents 1 mouse. (**G** and **H**) *n* = 486 MNs (WT, P50), 377 MNs (mSOD1, P50); 512 MNs (WT, P90), 411 MNs (mSOD1, P90). (**I**) *n* = 598 MNs (WT), 558 MNs (Srf KO), 584 MNs (mSOD1), and 650 MNs (mSOD1/Srf KO). Statistical testing was performed by 1-way ANOVA with Tukey corrections. Scale bar: 30 μm (**A**–**F**). *P < 0.05, **P < 0.01, ***P < 0.001.

First, we explored SRF expression across vulnerable and nonvulnerable MNs in WT and SOD1^G93A^ mice (henceforth mSOD1) in the lumbar (L3–L5) spinal cord (Figure 1). Corresponding to the presymptomatic and early symptomatic stages (37), we focused on the time points P50 and P90, respectively. Sections were immunostained for SRF together with ChAT (marker of all MNs) and MMP-9 (2, 15). SRF displayed a strong nuclear MN immunolocalization in both genotypes, with more than 50% of MNs displaying SRF immunostaining at P50 (Figure 1, A, B, and G). However, at P90 (Figure 1, C and D) — a time point after which degeneration of MN subpopulations has already occurred — a decrease in SRF^+^ MNs was observed in mSOD1 compared with WT mice (Figure 1G). Besides MNs (blue in Figure 1, A–F), SRF was also present in ChAT and VAChT negative neurons (green only in Figure 1, A–F).

Next, we analyzed SRF expression in vulnerable (MMP9^+^) and nonvulnerable (MMP9^–^) MNs at both P50 and P90 (Figure 1H). Here, approximately 60% of vulnerable and 40% of resistant MNs (MMP9^–^) were SRF^+^ (Figure 1H). The number of vulnerable and nonvulnerable SRF^+^ neurons was comparable in the WT mice (P50; Figure 1, G and H). However, in mSOD1 mice, more vulnerable neurons were SRF^+^ (approx. 70%; Figure 1H). At P90 (Figure 1, C and D), a decrease in SRF^+^ vulnerable MNs was observed in mSOD1 mice compared with P50 (Figure 1H). This downregulation between P50 and P90 was not observed to the same extent in WT mice (Figure1H).

In addition to mouse MNs, we analyzed the expression of SRF and its cofactor MRTF-A in MNs of sporadic human ALS cases (Supplemental Figure 1 and Supplemental Table 1; supplemental material available online with this article; https://doi.org/10.1172/jci.insight.167694DS1). In MNs of control cases, approximately 50% of all MNs had either nuclear SRF or MRTF-A localization (Supplemental Figure 1). In ALS patient–derived sections, we observed a lower number of SRF positive neurons, with approximately 20% of MNs being SRF^+^ (Supplemental Figure 1M). Although rarely observed in the less severe and later affected thoracic MNs of ALS cases, we identified a subpopulation of surviving MNs in the lumbar sections (up to 60%) displaying atypical nuclear SRF localization (Supplemental Figure 1N). These neurons revealed a rod-like or aggregated SRF nuclear morphology in contrast to the uniform SRF expression observed in control MNs (Supplemental Figure 1C vs. Supplemental Figure 1F). Conversely, no differences for MRTF-A between healthy samples and ALS specimens were observed (Supplemental Figure 1).

Taken together, SRF is present in both vulnerable rodent and human MNs, and a disruption of uniform SRF nuclear localization may be occurring in human ALS MNs.

### SRF deletion in MNs results in earlier disease onset in the SOD1 mouse model.

In order to investigate whether SRF executes a critical role in the vulnerability of MN subpopulations, we removed SRF from MNs in the context of the ALS-related SOD1^G93A^ mutation. First, S*rf^loxp/loxp^* mice were bred with *ChAT^Cre^* mice to obtain a line in which SRF was selectively deleted from MNs (and other cholinergic cells). The resulting S*rf^loxp/loxp;^ ^ChAT-Cre^* mice (henceforth called Srf KO) were viable and did not display any obvious phenotype. Subsequently, female S*rf^loxp/loxp; ChAT-Cre^* mice were bred with male mSOD1 mice, resulting in *hSOD1^G93A^/*S*rf^loxp/loxp;^ ^ChAT-Cre^* offspring (henceforth referred to as mSOD1/Srf KO). These mice were viable but were born at a frequency slightly lower than the expected mendelian rate of 6.25% (male: 4.74% and female 1.25%). SRF expression was abolished in MNs of the Srf-KO mice, but was detectable in non-MN cells of the spinal cord (Figure 1, E, F, and I). The efficiency of SRF ablation at P90 was identical in Srf-KO and mSOD1/Srf-KO animals (Figure 1I). At P50 and P90, downregulation of SRF-mediated gene expression was confirmed by a reduction in the SRF target gene c-Fos in MNs of mSOD1/Srf-KO compared with mSOD1 mice (Supplemental Figure 2). It is worth noting that the number of c-Fos^+^ MNs was induced in mSOD1 mice compared with WT (Supplemental Figure 2). Furthermore, vulnerable MMP9^+^ MNs colocalized more frequently with c-Fos compared with MMP9^–^ MNs in mSOD1 mice (Supplemental Figure 2).

We subjected mice of the 4 genotypes (WT, Srf-KO, mSOD1, and mSOD1/Srf-KO) to an initial behavioral assessment (exploratory cohort; Supplemental Figure 3), with body weight and grip strength measured separately in males and females. Interestingly, we found that male mSOD1/Srf-KO mice had a significantly lower body weight and reduced grip strength at P63 and P70–P77 than mSOD1 mice, implying a deterioration of the disease upon SRF loss in MNs.

**Figure 2:**
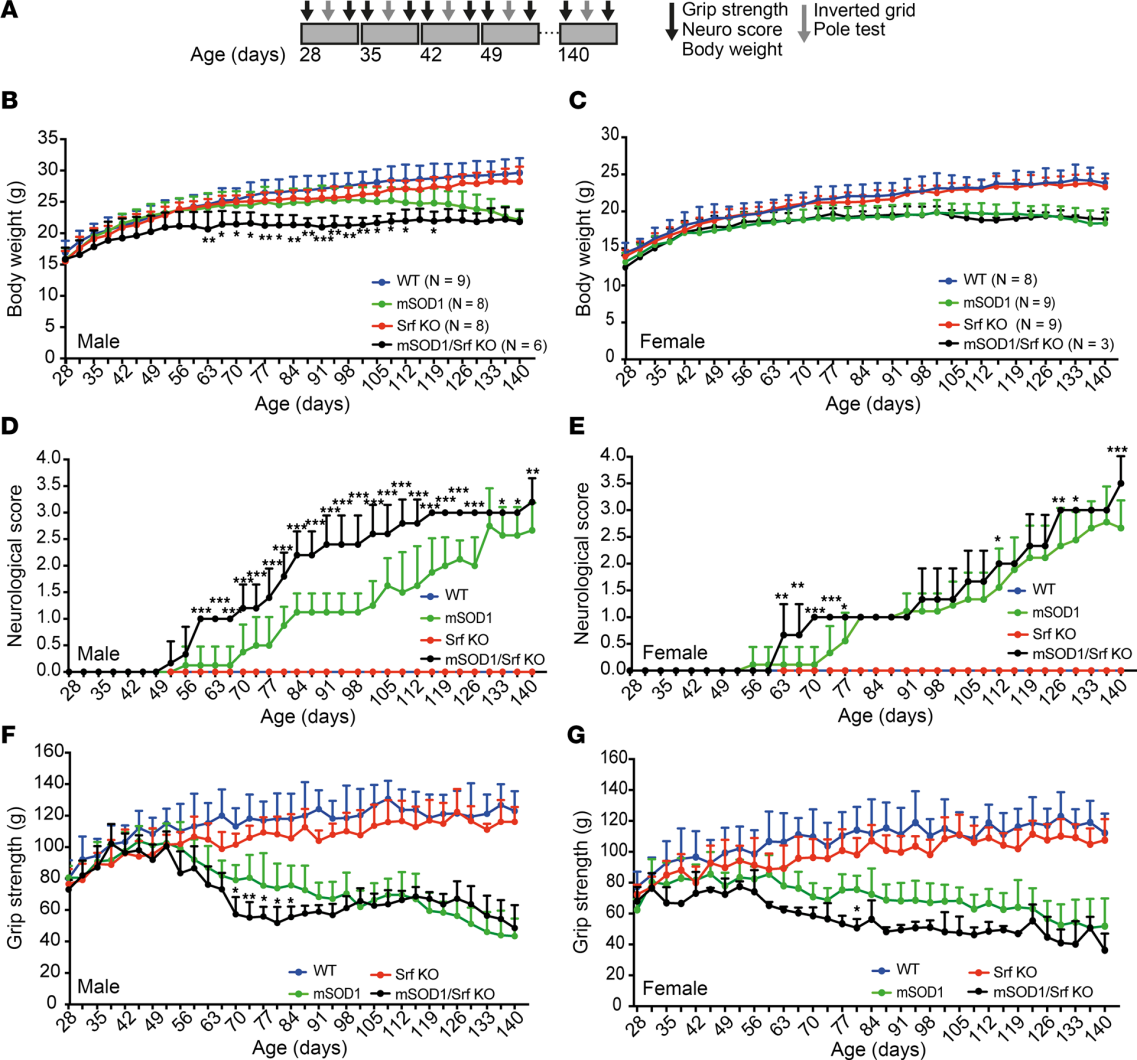
SRF deletion results in earlier disease onset in the SOD1 mouse model. (**A**) Animals were analyzed weekly from 28 to 140 days of age. In each week, grip strength, NeuroScore, and body weight were determined twice, and inverted grid and pole tests were performed once. (**B** and **C**) In male mSOD1/Srf-KO mice (**B**) a decrease in body weight was observed earlier compared with mSOD1 mice. This was not observed in female mice (**C**). (**D** and **E**) The NeuroScore showed an earlier elevation in male mSOD1/Srf-KO mice compared with mSOD1 mice (**D**). A similar phenotype was observed in female mSOD1/Srf-KO mice (**E**). (**F** and **G**) The grip strength of the forelimbs was decreased in both male (**F**) and female (**G**) mSOD1/Srf-KO mice compared with mSOD1 mice starting at approximately 8 weeks. *n* values are indicated in **B** and **C**. Statistical testing was performed by 2-way ANOVA with Tukey corrections. *P < 0.05, **P < 0.01, ***P < 0.001.

**Figure 3:**
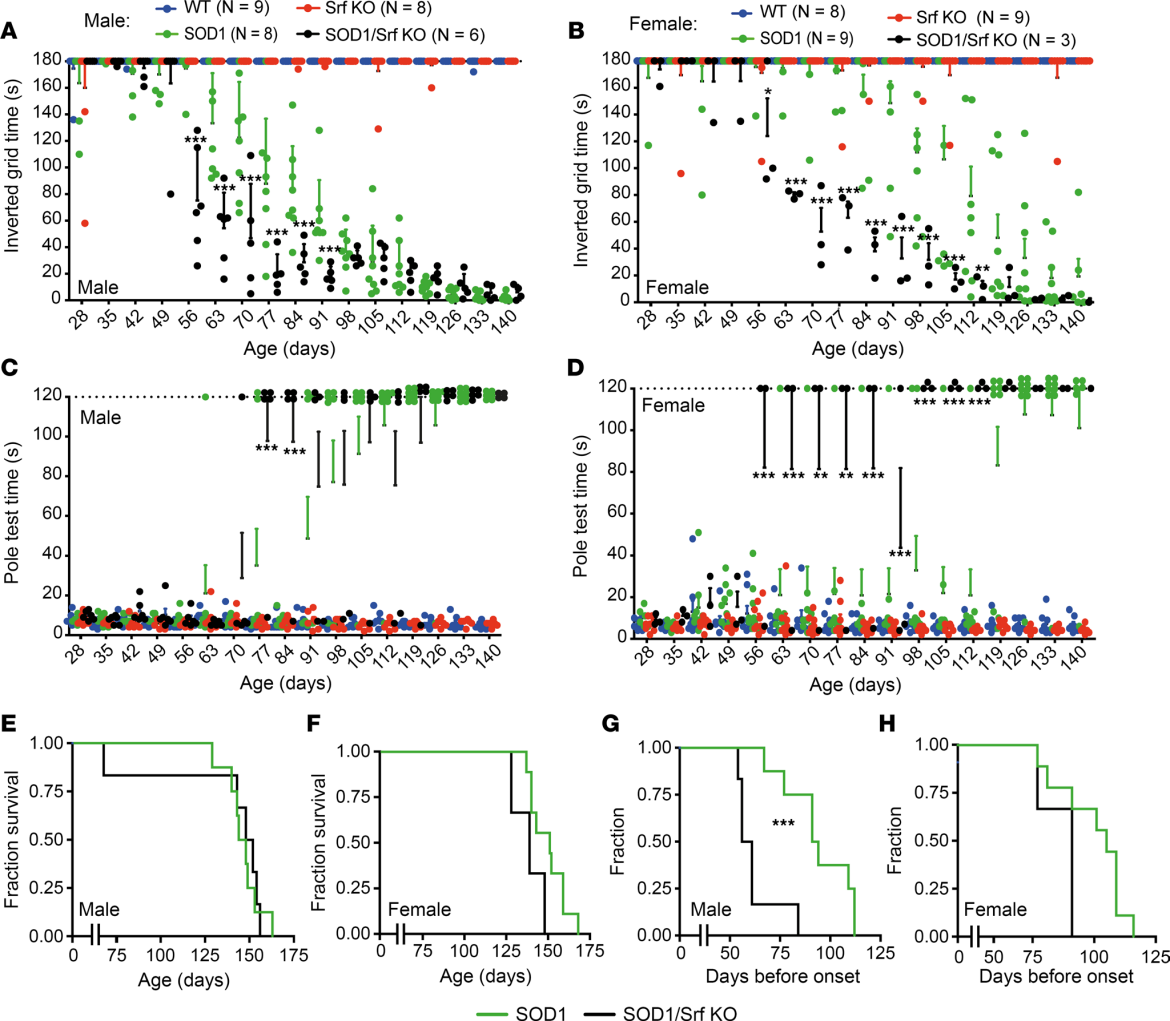
SRF deletion results in earlier motor impairments in SOD1 mice. (**A** and **B**) Male (**A**) and female (**B**) mSOD1/Srf-KO mice dropped down earlier in the inverted grid test compared with mSOD1 mice. WT and Srf-KO mice held on for 180 seconds. (**C** and **D**) In the pole test, male (**C**) and female (**D**) mSOD1/Srf-KO mice could not climb down the pole as quickly as mSOD1 mice. In contrast, WT and Srf-KO mice climbed down within 10 seconds. These data are presented as mean ± SEM. (**E** and **F**) The survival of male (**E**) or female (**F**) mSOD1/Srf-KO mice was comparable with mSOD1 mice. (**G** and **H**) The disease onset calculated on the basis of the body weight curves was earlier in male mSOD1/Srf-KO mice compared with mSOD1 mice (**G**). In female mice, the same tendency was observed (**H**). In **A**–**D**, each colored dot represents 1 mouse. *n* values are indicated in **A** and **B**. Statistical testing was performed by 2-way ANOVA with Tukey corrections (**A**–**D**) and Mantel-Cox test (**E**–**H**). **P < 0.01, ***P < 0.001.

These findings prompted us to explore the clinical, motor, and behavioral phenotype in a second, independent group of mice (in-depth cohort; Figures 2 and 3). The animal numbers in the exploratory and in-depth cohort were, respectively, as follows: for females, 14 and 8 WT, 10 and 9 Srf-KO, 14 and 9 mSOD1, and 11 and 3 mSOD1/Srf-KO, and for males, 13 and 9 WT, 8 and 8 Srf-KO, 8 and 12 mSOD1, and 8 and 6 mSOD1/Srf-KO animals. The reduced mendelian frequency obtained for female mSOD1/Srf-KO mice resulted in the inclusion of fewer female compared with male animals. To analyze the in-depth cohort, we considered multiple ALS-associated phenotypes including the neurological score (NS) and body weight (37, 38), grip strength, inverted grid and pole test (Figure 2 and Figure 3), and overall survival (Figure 3). Since there is a substantial sex effect in ALS (39), male and female mice were investigated separately.

In the in-depth cohort (Figures 2 and 3), male SOD1 animals exhibited a plateau in weight gain at the age of 10 weeks, followed by a subsequent decline in weight starting around 13–14 weeks (Figure 2B). In contrast, WT and Srf-KO groups displayed steady weight gain (Figure 2B). Interestingly, male mSOD1/Srf-KO mice stopped gaining weight before the mSOD1 mice, reached a lower peak body weight, and started losing weight already at 8–9 weeks (Figure 2B). In contrast to males, there was no obvious difference in the body weight of mSOD1/Srf-KO females when compared with the SOD1 females (Figure 2C).

mSOD1 and mSOD1/Srf-KO mice also showed differences in their NeuroScore assessments (37, 40). Male mSOD1 mice became symptomatic (NeuroScore 1) at around 12–13 weeks of age and reached a maximum severity score of 2.5 by week 20 (Figure 2D). In contrast, mSOD1/Srf-KO mice reached NeuroScore 1 much earlier at 8 weeks and NeuroScore 3 at 16 weeks (Figure 2D). Furthermore, the NeuroScore of mSOD1/Srf-KO mice was significantly higher than in mSOD1 mice in most time points after 8 weeks. Female mSOD1/Srf-KO animals also displayed an earlier appearance of symptoms (NeuroScore 1), but the magnitude was smaller (Figure 2E).

We employed grip measurement (Figure 2, F and G) and the inverted grid test (Figure 3, A and B) to assess grip strength. In mSOD1 mice of both sexes, grip strength began to diverge from WT and Srf-KO mice at the age of 7–8 weeks (1, 6). Loss of SRF in MNs of the mSOD1/Srf-KO cohort resulted in a more pronounced decrease in grip strength, which was evident at the age of 8 weeks. However, by the age of 15 weeks, the male mSOD1/Srf-KO mice were no longer different from male mSOD1 mice (Figure 2F). A similar trend was observed for female mice, although the lower sample numbers precluded a higher statistical significance (Figure 2G).

In the inverted grid test (Figure 3, A and B), male and female mSOD1 mice were no longer able to complete the test by the age of 8 weeks (falling down before 180 seconds). At the same age, mSOD1/Srf-KO mice displayed shorter holding times than mSOD1 animals (Figure 3, A and B), indicating a more severe motor impairment in alignment with the grip strength results (Figure 2, F and G). In contrast, WT and Srf-KO mice adhered to the grid for ≥ 180 seconds over the entire 16-week period.

Moreover, we assessed mice for motor coordination in the pole climb-down test (Figure 3, C and D). Here, most of the mSOD1 mice were no longer able to climb down the pole at the age of 13 weeks. mSOD1/Srf-KO mice were unable to climb down the pole at a significantly earlier age and, when capable, required a significantly longer time than mSOD1 mice. WT and Srf-KO mice were consistently able to complete the task.

Finally, we characterized the disease onset based on the body weight curves (Figure 2, B and C), determining the presymptomatic (onset) phase and the progression phase durations (38). SRF ablation in males (Figure 3G) but not in females (Figure 3H) resulted in a significantly earlier onset of disease, although the duration of the progression phase remained unaffected. Surprisingly, despite the earlier onset of symptoms, no difference was found between mSOD1 and mSOD1/Srf-KO mice (either male or female) in relation to their survival (Figure 3, E and F).

Taken together, loss of SRF in MNs causes an earlier disease onset and a more profound initial motor impairment in male and — to some extent — female mice. Surprisingly, this finding did not align with a shorter survival, indicating that SRF plays a role in determining the vulnerability of a subset of MNs that are affected first (the FF-MN).

### SRF deletion in MNs enhances microglial reactivity and denervation of NMJs.

**Figure 4:**
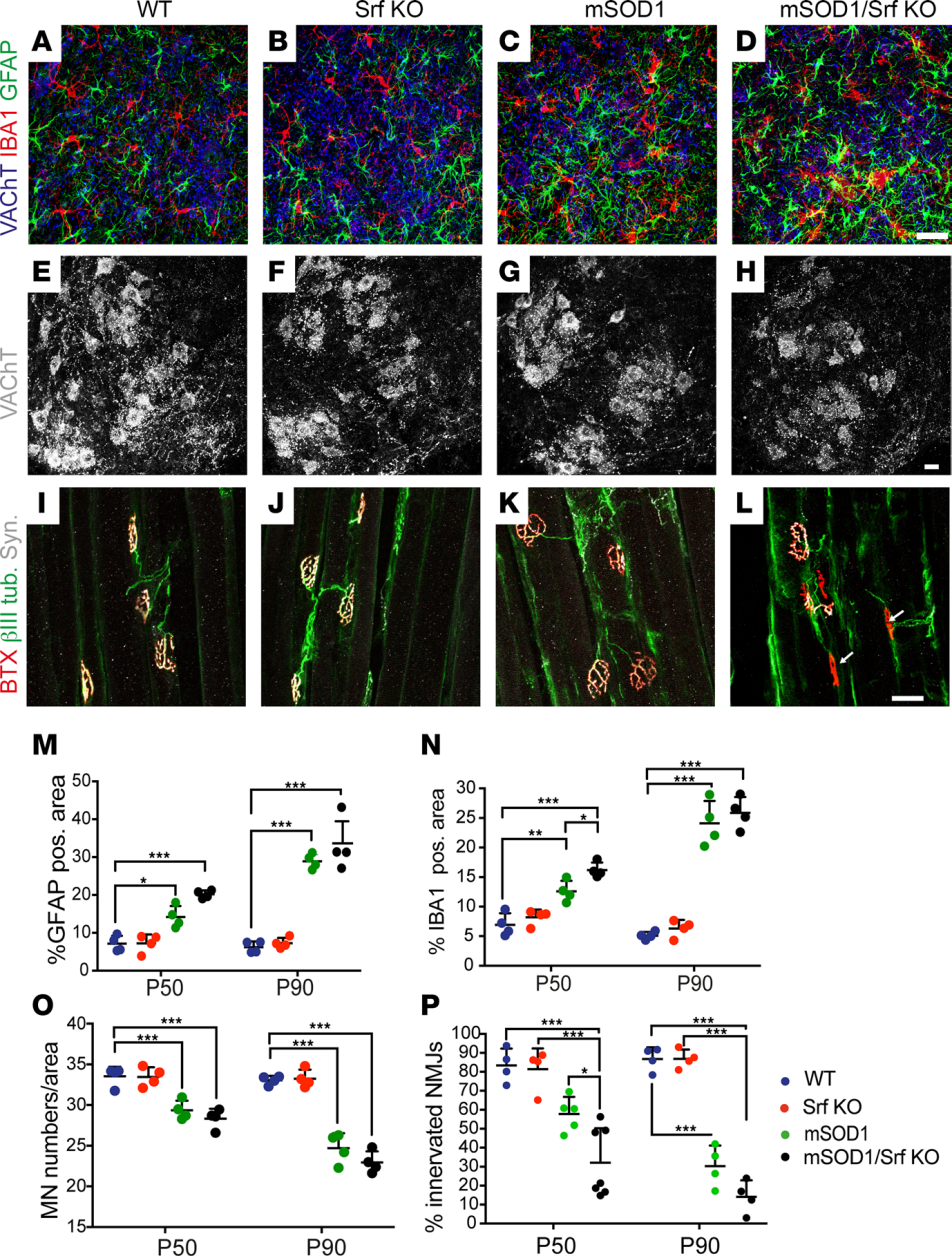
SRF deletion enhances microgliosis and accelerates neuromuscular junction denervation. (**A–D**) Ventral horns from P50 WT (**A**), Srf-KO (**B**), mSOD1 (**C**), and mSOD1/Srf-KO (**D**) mice were stained for VAChT (blue), microglia (IBA1; red), and astrocytes (GFAP; green). In mSOD1/Srf-KO mice, microglia and less pronounced astrocyte abundance was elevated compared with other cohorts (see **M** and **N**). (**E**–**H**) The MN number in ventral horns labeled with VAChT was reduced in mSOD1 (**G**) and mSOD1/Srf-KO (**H**) compared with WT (**E**) and Srf-KO (**F**) mice. However, no differences between mSOD1 (**G**) and mSOD1/Srf-KO (**H**) cohorts were discernible. (**I**–**L**) In mSOD1/Srf-KO mice (**L**), a reduction in NMJ innervation was observed (see NMJs marked with arrows). (**M** and **N**) Quantification of GFAP (**M**) and IBA1 (**N**) abundance in P50 and P90 mice. (**O**) The MN number was reduced in mSOD1 and mSOD1/Srf-KO cohorts at P50 and more pronounced at P90 compared with WT and Srf-KO mice. (**P**) In mSOD1/Srf-KO mice, the percentage of innervated NMJs was decreased significantly at P50 and with the same tendency at P90 in relation to mSOD1 mice. In **M**–**P**, each colored dot represents 1 mouse. In **O**, *n* of MNs/sections analyzed were for P50 as follows: 1,483/44 (WT); 1,713/51 (Srf KO); 1,484/50 (mSOD1); 1,428/50 (mSOD1/Srf KO). For P90: 1,322/40 (WT); 1330/40 (Srf KO); 988/40 (mSOD1); 940/41 (mSOD1/Srf KO). In **P**, *n* for NMJ were for P50 as follows: 462 (WT), 432 (Srf KO), 472 (mSOD1), 834 (mSOD1/Srf KO). *n* for NMJ were for P90 as follows: 456 (WT), 458 (Srf KO), 480 (mSOD1), 441 (mSOD1/Srf KO). Statistical testing was performed by 1-way ANOVA with Tukey corrections. Scale bar: 30 μm. *P < 0.05, **P < 0.01, ***P < 0.001.

Next, we investigated the histopathological basis of the earlier and more severe disease onset in mSOD1/Srf-KO mice (Figure 4). More specifically, we assessed microgliosis and astrogliosis in the ventral horn of the lumbar spinal cord (Figure 4, A–D). WT and Srf-KO mice were comparable in astrocyte and microglia density (Figure 4, A and B). mSOD1 mice exhibited marked astrogliosis and microgliosis, which were significant at P50 and further intensified at P90 (Figure 4, C, M, and N). Notably, at P50 — but not P90 — mSOD1/Srf-KO mice displayed a higher degree of microgliosis compared with mSOD1 littermates and a tendency for higher astrogliosis (Figure 4, D, M, and N). This was corroborated by an additional marker CD68, indicating microglia-associated phagocytosis (Supplemental Figure 4). Overall, there was a slight increase in microgliosis and astrogliosis at P50, but not P90, when SRF was deleted in mSOD1 mice.

Furthermore, quantification of MNs (Figure 4O) revealed reduced MN numbers in mSOD1 (Figure 4G) and mSOD1/Srf-KO (Figure 4H) mice compared with WT (Figure 4E) and Srf-KO (Figure 4F). However, no differences between mSOD1 and mSOD1/Srf-KO mice were discernible (Figure 4O).

Finally, we scrutinized the extent of NMJ denervation (Figure 4, I–L, and P). SRF loss appears to primarily affect the early disease phase, when FF-MN denervate and, subsequently, degenerate. Thus, we focused on the L1 subcompartment of the *lateral gastrocnemius*, containing only FF motor units denervating at about P55 (1, 6, 18). To label the presynaptic terminals and the axons, muscle sections were immunostained for synaptophysin and βIII tubulin, respectively. Thereafter, sections were stained with fluorescently labeled bungarotoxin (BTX) to identify the postsynaptic NMJ plaques (Figure 4, I–L). NMJs were considered “innervated” if the overlap between the pre- and postsynaptic markers was > 70% of the NMJ area. In this respect, both WT and Srf-KO mice displayed > 80% fully innervated NMJs. mSOD1 mice displayed a trend toward a decrease in L1 innervation (Figure 4P). However, by P50, the mSOD1/Srf-KO mice exhibited a significant degree of NMJ denervation, once again indicating an earlier disease onset (arrows Figure 4, L and P). At P90, mSOD1/Srf-KO animals still displayed a trend toward more severe denervation than in mSOD1 mice (Figure 4, L and P).

Thus, disease markers show an earlier and mildly enhanced microgliosis and NMJ denervation in mSOD1/Srf-KO mice.

### Reduced autophagy induction and enhanced misfolded SOD1 accumulation upon SRF deletion in SOD1^G93A^ mice.

Autophagy induction is one of the cellular hallmarks described in several ALS mouse models, including SOD1^G93A^ mice (19, 37, 41) and human MNs (42). Furthermore, autophagy impairment has previously been hypothesized to be mechanistically related to MN vulnerability (43).

**Figure 5:**
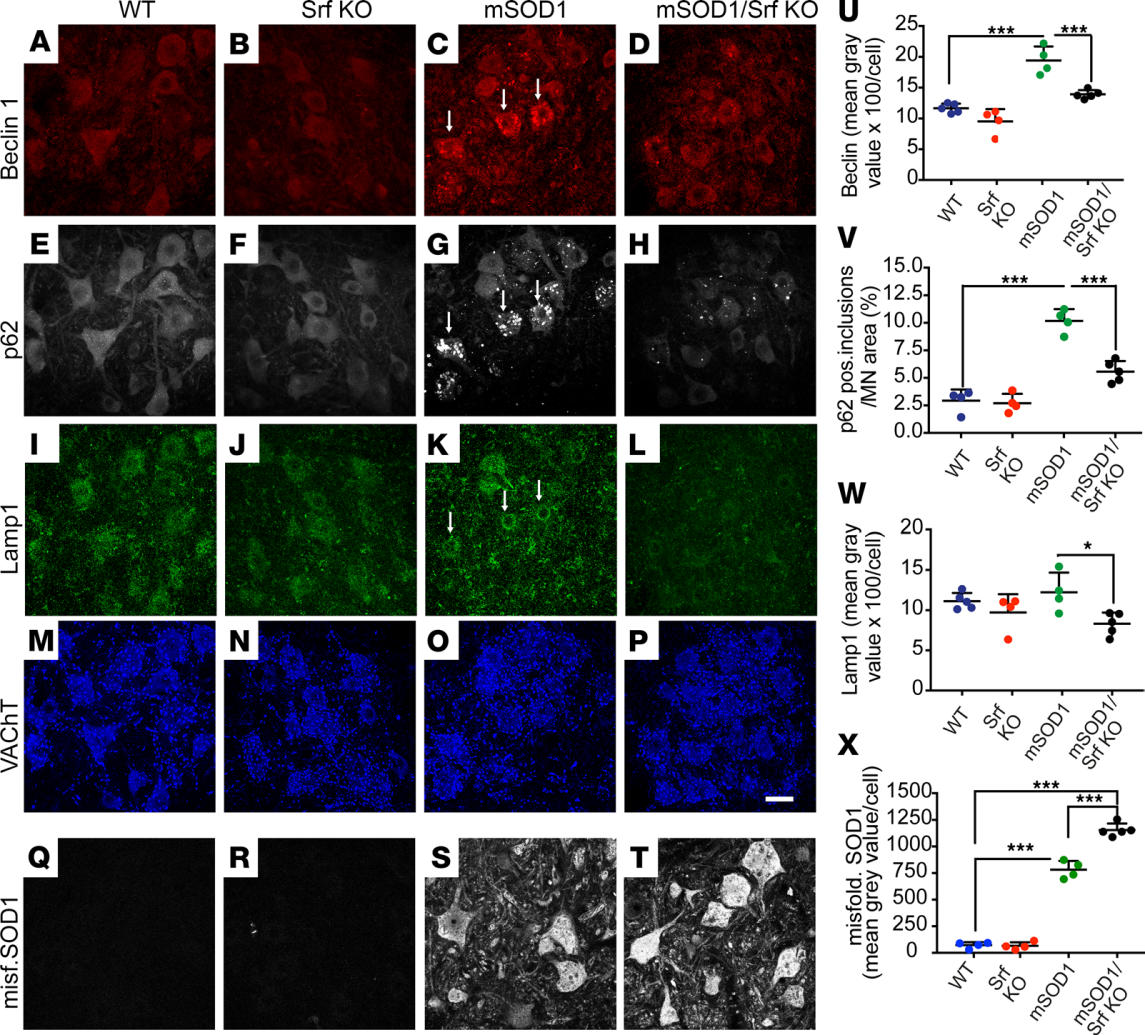
Reduced autophagy induction upon SRF deletion in SOD1 mice. (**A**–**D**) Beclin1 abundance was upregulated in P50 ventral horns of mSOD1 (arrows in **C**) compared with WT (**A**) and Srf-KO (**B**) mice. SRF deletion in SOD1 mice (**D**) reduced the Beclin1 abundance. (**E**–**H**) p62 was upregulated in MNs of mSOD1 mice (arrows in **G**). In mSOD1/Srf-KO mice, p62 accumulation was diminished (**H**). (**I**–**L**) Lamp1 was more abundant in mSOD1 mice (arrows in **K**), and this was reduced in mSOD1/Srf-KO (**L**) mice. (**M**–**P**) VAChT staining of all 4 cohorts in corresponding regions. (**Q**–**T**) Misfolded SOD1 was absent from WT (**Q**) and SRF deficient (**R**) MNs, whereas accumulations were found in mSOD1 (**S**) and more pronounced in mSOD1/Srf KO (**T**) mice. (**U**–**X**) Beclin1 (**U**), p62 (**V**), and Lamp1 (**W**) signals were induced in mSOD1 mice compared with WT mice. This was not observed to the same extent in mSOD1/Srf-KO animals. Misfolded SOD1 was present in mSOD1 (**S**) and stronger in mSOD1/Srf KO MNs (**T** and **X**). In **U**–**X**, *n* values are indicated by each colored dot reflecting 1 mouse. In **U**–**W**, *n* values for MNs analyzed were: 305 (WT), 293 (Srf KO), 287 (mSOD1), and 383 (mSOD1/Srf KO). In **X**, MN numbers were as follows: 619 (WT), 682 (Srf KO), 413 (mSOD1), 400 (mSOD1/Srf KO). Statistical testing was performed by 1-way ANOVA with Tukey corrections. Scale bar: 30 μm. *P < 0.05, ***P < 0.001.

Therefore, we analyzed whether SRF loss affects the autophagic pathway in MNs of the mSOD1 model (Figure 5). At P50, lumbar spinal cord sections were obtained from male mice representing the 4 genotypes (Figure 2, Figure 3, and Figure 4) and were immunostained for the early autophagy marker Beclin-1 (Figure 5, A–D, and U), the autophagy substrate p62 (Figure 5, E–H, and V), and the lysosomal marker Lamp1 (Figure 5, I–L, and W).

Compared with WT or Srf-KO littermates, mSOD1 mice displayed MNs with an increased burden of Beclin-1^+^ structures located in the cell body (Figure 5C). Surprisingly, in mSOD1/Srf-KO mice, very few MNs displayed high levels of Beclin-1^+^ aggregates, and Beclin-1 intensity in MNs was significantly lower than in mSOD1 mice (Figure 5, D and U). Similarly, a MN subset in mSOD1 mice displayed round inclusions that were brightly immunopositive for p62 (37, 41). Once again, in mSOD1/Srf-KO mice, MNs displaying p62 inclusions were rare and had smaller p62 inclusions (Figure 5, H and V). In fact, most MNs in mSOD1/Srf-KO mice were devoid of p62 inclusions (Figure 5, H and V). In line with the apparent decrease in the activity of the autophagic pathway following SRF loss, we found that the Lamp1 was not upregulated in mSOD1/Srf-KO mice (Figure 5, L and W) when compared with mSOD1 mice (arrows in Figure 5K). We also inspected LC3, a marker of the terminal stages of autophagy flux (Supplemental Figure 5). On a protein level, LC3 abundance was enhanced in mSOD1/Srf-KO mice compared with mSOD1 mice (Supplemental Figure 5). Above, we noted that lysosomal abundance analyzed by Lamp1 was decreased in mSOD1/Srf-KO mice (Figure 5, L and W). Thus, fusion of LC3^+^ autophagosomes with lysosomes in the final stages of autophagic flux might be impaired by SRF deletion, thereby resulting in the accumulation of LC3^+^ autophagosomes in mSOD1/Srf-KO mice.

Since SRF ablation appeared to interfere with autophagy-mediated protein degradation, the abundance of misfolded SOD1 was monitored (Figure 5, Q–T, and X). Interestingly, in line with reduced autophagy, the misfolded SOD1 burden (Figure 5S) was significantly elevated in mSOD1/Srf-KO (Figure 5T) compared with mSOD1 (Figure 5S) mice at P50 (quantified in Figure 5X).

SRF loss interferes with the activity of the autophagic pathway, and this observation correlated with a heavier burden of misfolded SOD1 in SRF-deficient MNs.

### SRF ablation reduces autophagy gene mRNA abundance in mutant SOD1 MNs.

**Figure 6:**
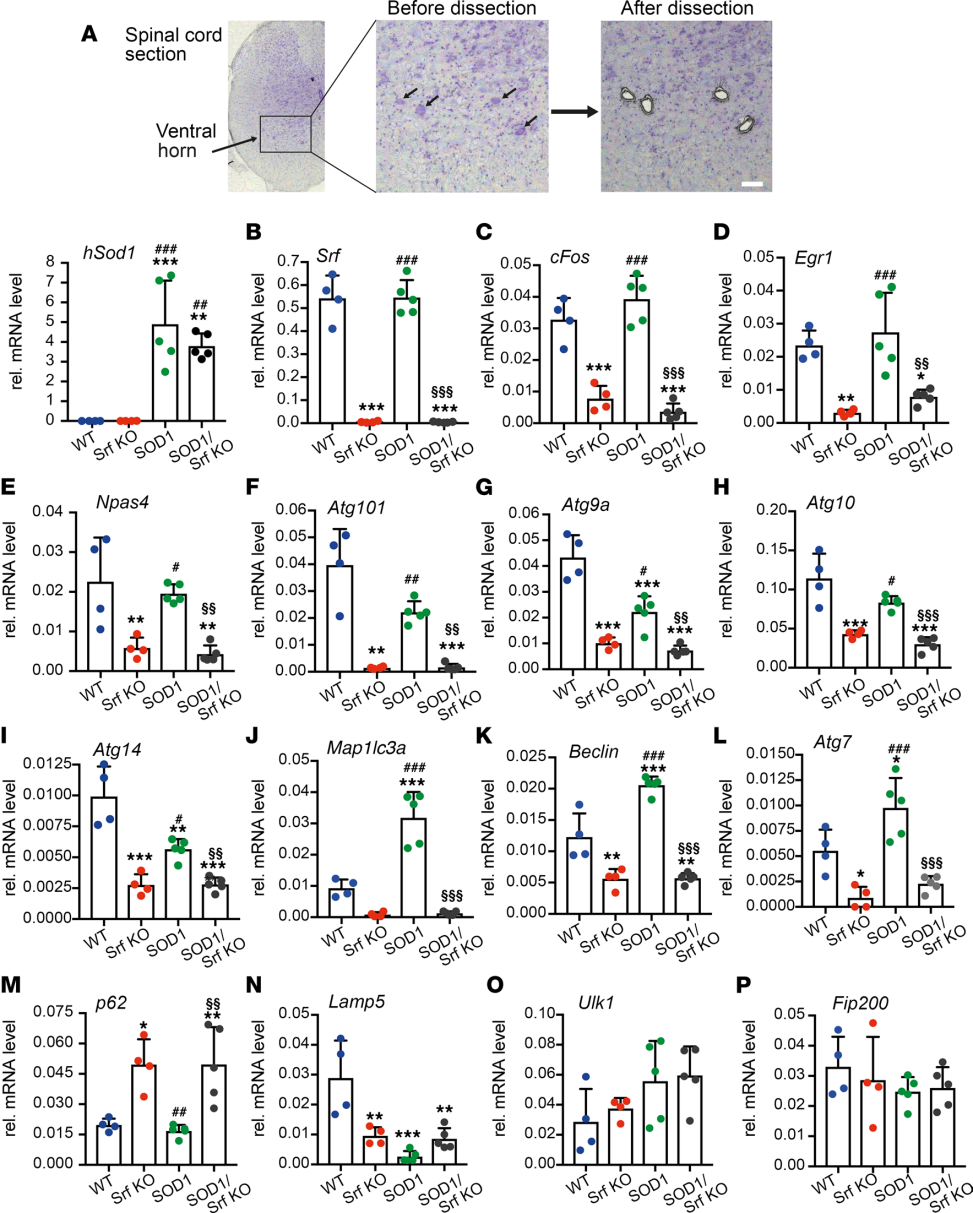
Reduced autophagy induction upon SRF deletion in SOD1 mice. (**A**) MNs from the ventral horn of P50 mice were subjected to qPCR analysis. Arrows point at MNs before dissection. After dissection, MNs were selectively removed. The mRNA abundance of human *Sod1* was specifically increased in MNs of mSOD1 and mSOD1/Srf-KO mice. (**B**) *Srf* mRNA was removed from MNs of Srf-KO and mSOD1/Srf-KO animals. (**C**–**E**) *cFos* (**C**), *Egr1* (**D**), and *Npas4* (**E**) were reduced in Srf-KO animals and to a similar extent in mSOD1/Srf-KO mice. (**F**–**I**) In relation to WT, mRNA levels of *Atg101* (**F**), *Atg9*a (**G**), *Atg10* (**H**), and *Atg14* (**I**) were reduced in Srf-KO and mSOD1 MNs. It is important to note that, in mSOD1/Srf KO MNs, the mRNA levels of all 4 genes were lower compared with mSOD1 MNs. (**J**–**L**) mRNA abundance of *Map1lc3a* (**J**), *Beclin1* (**K**), and *Atg7* (**L**) was reduced in Srf-KO and upregulated in mSOD1 MNs in relation to WT. The upregulation of all 3 genes in mSOD1 MNs was not observed in mSOD1/Srf-KO MNs. (**M**) *p62* mRNA was not induced in Srf-KO and mSOD1/Srf-KO MNs in relation to WT and mSOD1 MNs. (**N**) *Lamp5* abundance was downregulated in Srf-KO, mSOD1 and mSOD1/Srf-KO MNs in comparison with WT. (**O** and **P**) *Ulk1* (**O**) and *Fip200* (**P**) abundance was not overtly changed between cohorts. In **A**–**P**, *n* values are indicated by each colored dot reflecting 1 mouse. *, #, § denote significance in relation to WT, Srf-KO, and mSOD1, respectively. *P < 0.05, **P < 0.01, ***P < 0.001. Statistical testing was performed by 1-way ANOVA with Tukey corrections. Scale bar: 30 μm.

So far, SRF has not been revealed as a TF associated with autophagy in any cell type. However, given the impact of SRF loss on MN autophagy (Figure 5), we questioned whether SRF could affect transcription of genes encoding for the autophagy pathway (Figure 6). Thus, we microdissected individual MNs for mRNA extraction and cDNA synthesis in the lumbar ventral motor column in P50 male mice of the 4 genotypes (Figure 6). Quantitative PCR (qPCR) analysis confirmed comparable human *SOD1* transgene expression in the mSOD1 and mSOD1/Srf-KO cohort, with no *SOD1* mRNA detected in WT and Srf-KO mice (Figure 6A). *Srf* mRNA deletion was validated in Srf-KO and mSOD1/Srf-KO mice (Figure 6B). Furthermore, several IEGs known as SRF targets (25, 36), such as *cFos* (Figure 6C), *Egr1* (Figure 6D), or *Npas4* (Figure 6E), were reduced in Srf-KO and — to a similar extent — in mSOD1/Srf-KO mice, thus providing confirmation of an impairment in SRF-dependent transcription.

A first gene set including *Atg101*, *Atg9a*, *Atg10*, and *Atg14* (Figure 6, F–I) was downregulated in Srf-KO MNs. Albeit to a lesser extent, these genes were also downregulated in mSOD1 MNs in comparison with the WT. Of particular importance, these genes were even further decreased in mSOD1/Srf-KO MNs compared with mSOD1 MNs (Figure 6, F–I). A second gene set encompassing *Map1lc3a* (i.e., *Lc3a*), *Beclin1*, and *Atg7* (Figure 6, J–L) showed a substantial mRNA upregulation indicative of autophagy induction in mSOD1 samples; however, their expression was downregulated in Srf-KO mice and abrogated in mSOD1/Srf-KO MNs. These data indicate impaired autophagy induction upon SRF deletion due to reduced mRNA levels of autophagy genes. We also analyzed p62, a gene that has previously been investigated on the protein level (Figure 5). Here, p62 levels were upregulated in SRF-deficient MNs and mSOD1/Srf-KO MNs, suggesting a SRF repressor function in WT MNs (Figure 6M). *Lamp5* was downregulated in Srf-KO, mSOD1, and mSOD1/Srf-KO MNs in comparison with WT (Figure 6N). Two further autophagy-encoding genes, *Ulk1* and *Fip200*, were not obviously affected (Figure 6, O and P). To assess direct SRF promoter occupancy, ChIP was performed (Supplemental Figure 6). *Atg7*, *Atg9a*, and *Atg10* were predicted to contain SRF binding sites (CArG boxes; Supplemental Table 2), and enhanced SRF promoter occupancy for those genes was observed (Supplemental Figure 6).

In summary, SRF loss affects transcriptional regulation of multiple autophagy genes, and autophagy induction in ALS MNs requires SRF.

### SRF-VP16 reduces neurodegeneration-associated induction of autophagic genes.

Previously, we noted decreased induction of autophagy-related proteins (Figure 5) and mRNA abundance (Figure 6) upon SRF loss of function. To corroborate SRF’s transcriptional regulation on autophagy-encoding genes, we performed gain-of-function experiments with SRF-VP16, a constitutively active SRF protein (44). As a control, SRF-VP16ΔMADS was used that did not contain the MADS box, thereby precluding DNA binding (44). Considering that the accumulation of misfolded proteins induces autophagy, we further asked whether SRF-VP16 reduces misfolded protein abundance triggered by autophagy induction. For this purpose, neurodegeneration-associated autophagy was induced by Poly-GA aggregates recapitulating C9orf72-associated ALS pathological features (20).

**Figure 7:**
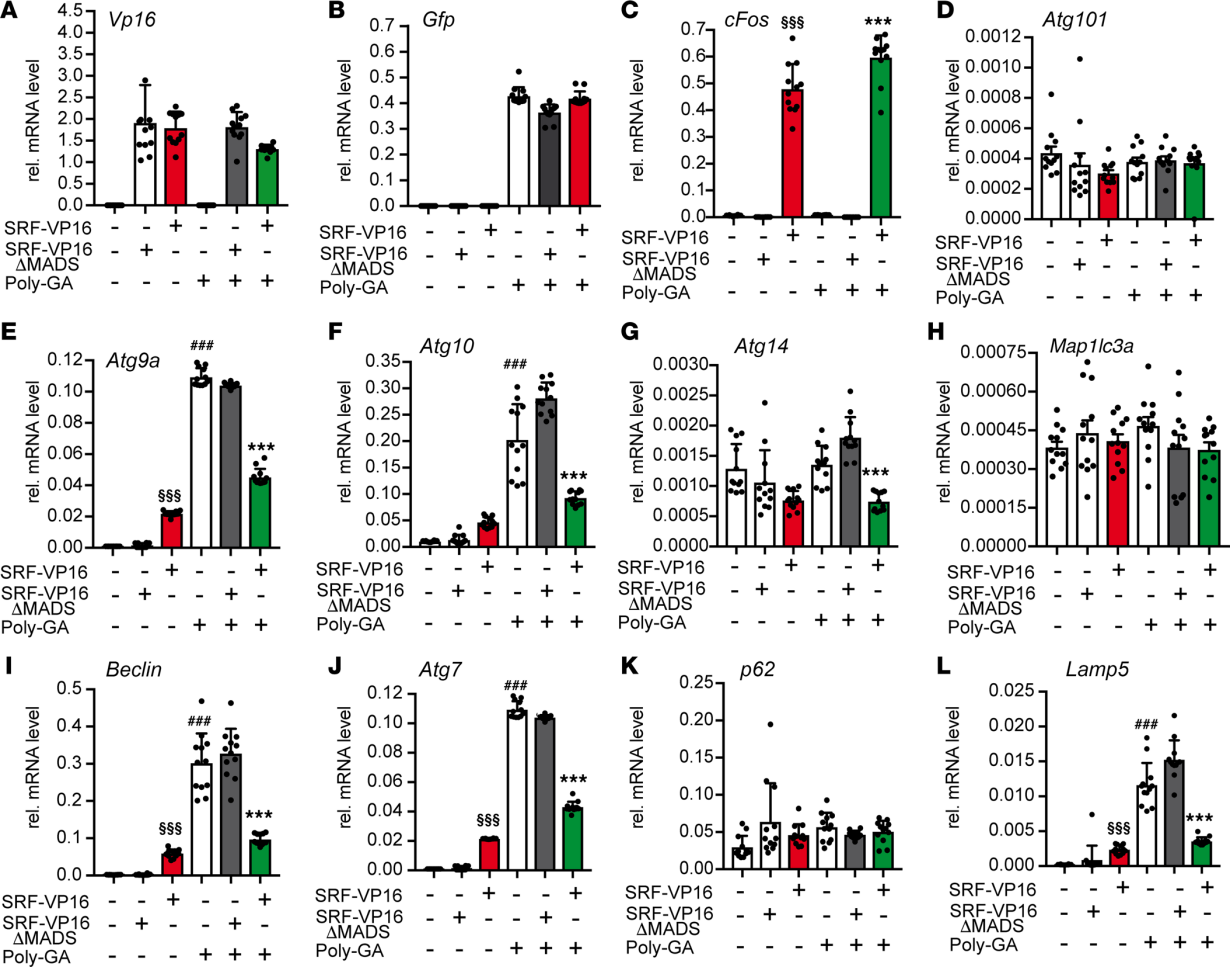
SRF-VP16 reduces C9orf72-associated induction of autophagy genes. HEK293 cells expressed either constitutively active SRF-VP16 or inactive SRF-VP16ΔMADS in the presence or absence of aggregates formed by Poly-GA expression. Subsequently, qPCR was performed to assess mRNA abundance of the genes indicated. (**A**–**C**) Expression of *Vp16* tagged SRF (**A**) and *Gfp* tagged Poly-GA (**B**) was similar on an mRNA level. SRF-VP16 but not SRF-VP16ΔMADS induced *cFos* (**C**). (**D**–**L**) SRF-VP16 but not SRF-VP16ΔMADS induced *Atg9a* (**E**), *Atg10* (**F**), *Beclin* (**I**), and *Atg7* (**J**) as well as *Lamp5* (**L**). Poly-GA expression upregulated *Atg9a* (**E**), *Atg10* (**F**), *Beclin* (**I**), *Atg7* (**J**), and *Lamp5* (**L**) but not *Atg101* (**D**), *Atg14* (**G**), *Map1lc3a* (**H**), and *p61* (**K**). SRF-VP16 but not SRF-VP16ΔMADS downregulated several of those autophagy- and lysosome-encoding genes induced by Poly-GA aggregate formation. In **A**–**L**, *n* values are indicated by each black dot reflecting 1 cell culture dish. *, #, § denote significance between SRF-VP16 and SRF-VP16ΔMADS in the presence of Poly-GA (fifth and sixth bar), between mock and Poly-GA (first and fourth bar), and between SRF-VP16 and SRF-VP16ΔMADS (second and third bar), respectively. *P < 0.05, **P < 0.01, ***P < 0.001. Statistical testing was performed by 1-way ANOVA with Tukey corrections.

An equal abundance of SRF-VP16 or SRF-VP16ΔMADS mRNA (Figure 7A) and GFP-tagged Poly-GA aggregates was documented by qPCR (Figure 7B). As expected, SRF-VP16 — but not SRF-VP16ΔMADS — induced the SRF target gene *cFos* (Figure 7C).

In total, we tested 8 autophagy-encoding (Figure 7, D–K) and 1 lysosomal pathway–encoding gene (*Lamp5*; Figure 7L). SRF-VP16, but not SRF-VP16ΔMADS, induced 4 of the 8 autophagy-encoding genes, including *Atg9a*, *Atg10*, *Beclin1*, and *Atg7* as well as *Lamp5*. Moreover, all genes upregulated by Poly-GA expression, *Atg9a*, *Atg10*, *Atg14*, *Beclin1*, *Atg7*, and *Lamp5* (Figure 7) were downregulated by SRF-VP16. This suggests that SRF-VP16 exerts transcriptional activation under typical physiological cell conditions, whereas SRF-VP16 acts as transcriptional repressor in the context of a neurodegenerative disease–inducing protein. Conversely, in the presence of SRF-VP16ΔMADS, Poly-GA aggregate–mediated transcriptional induction persisted. Other genes, including *Atg101* (Figure 7D), *Map1lc3a* (Figure 7H), and *p62* (Figure 7K), were not obviously modulated by either SRF-VP16 or Poly-GA expression.

Besides SRF-VP16, we additionally analyzed whether MRTF-A, an SRF cofactor with considerable neuronal functions (20), also mediates transcriptional regulation of autophagy genes in the context of Poly-GA–inflicted aggregate formation (Supplemental Figure 7). MRTF-A was also found to upregulate the same set of autophagy- and lysosome-encoding genes (Supplemental Figure 7). Furthermore, Poly-GA–mediated induction of the aforementioned genes was also counteracted by MRTF-A (Supplemental Figure 7). Besides Poly-GA, we analyzed whether SRF-VP16 can reduce autophagy-encoding genes induced by mutant SOD1 proteins, resulting in aggregate formation (SOD1^G93A^ and SOD1^A4V^) (45). SRF-VP16 reduced the mRNA abundance of *Atg9a*, *Atg10*, *Atg7*, and *Lamp5*, which were induced by both SOD1^G93A^ and SOD1^A4V^ (Supplemental Figure 8).

In summary, SRF-VP16 can dampen autophagy induction prompted by an established ALS-associated protein aggregate.

### SRF-VP16 modulates autophagy and reduces ALS-associated aggregate formation.

**Figure 8:**
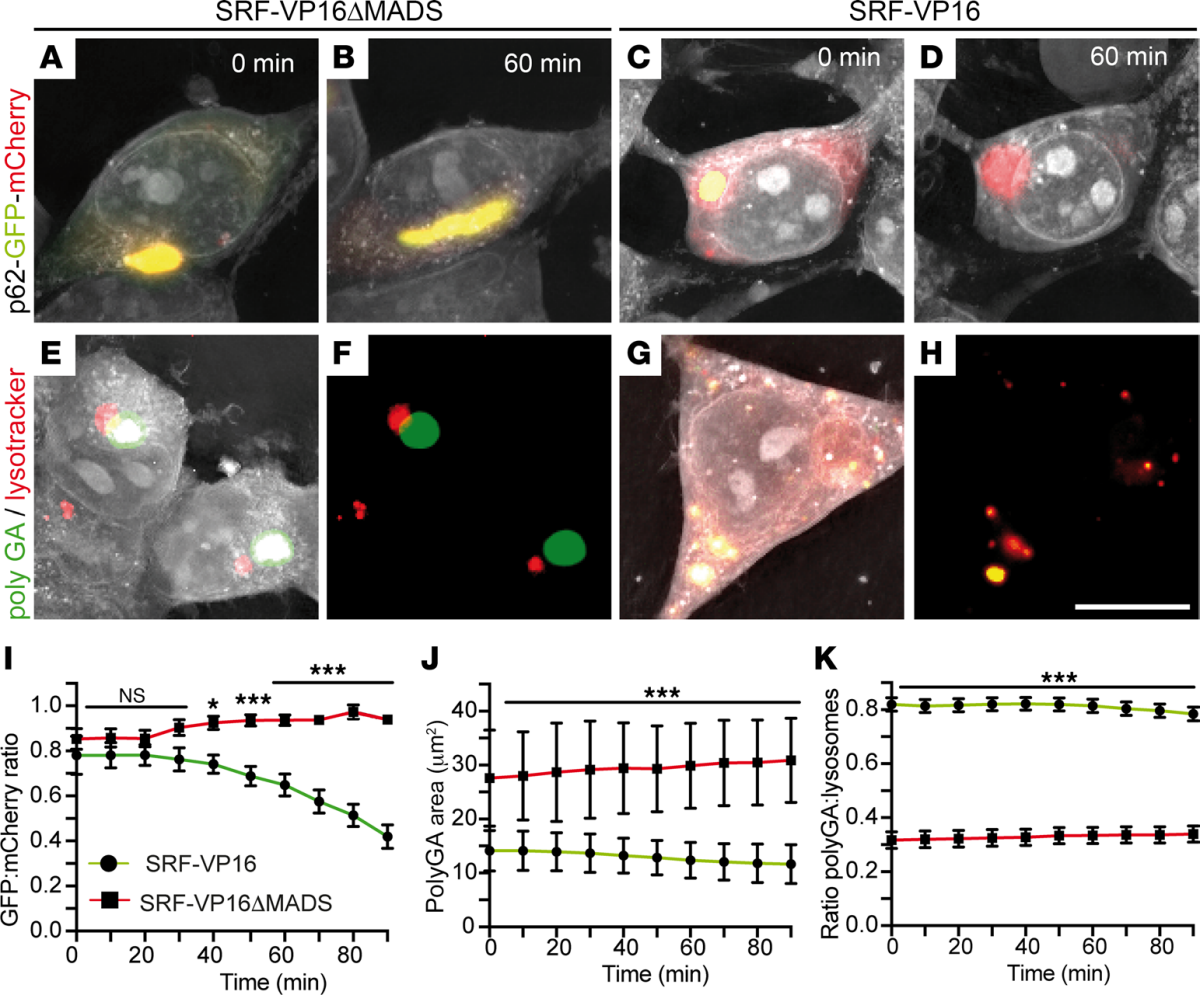
SRF-VP16 enhances autophagy Poly-GA aggregate clearance. (**A**–**D**) HEK293 cells transfected with a p62-GFP-mCherry construct coexpressed either SRF-VP16ΔMADS (**A** and **B**) or SRF-VP16 (**C** and **D**). Cells were imaged over 90 minutes, and pictures show the starting time point (0 minutes; **A** and **C**) or 60 minutes (**B** and **D**). Enhanced autophagy is indicated by a color change from yellow to red. SRF-VP16 enhanced autophagy propagation as indicated by yellow vesicles at t = 0 (**C**) turning at t = 60 minutes into red (**D**). In SRF-VP16ΔMADS–expressing cells, the ratio between GFP/mCherry remained constant (**A** and **B**). (**E**–**H**) HEK293 cells expressing Poly-GA aggregates (green) were stained with lysotracker (red). SRF-VP16 (**G** and **H**), but not as much SRF-VP16ΔMADS (**E** and **F**), reduced aggregate size and enhanced colocalization of aggregates with lysosomes (yellow in **G** and **H**). (**I**) The GFP/mCherry ratio was decreased in SRF-VP16 compared with SRF-VP16ΔMADS–expressing cells, indicating enhanced autophagic flux by SRF-VP16 (*n* = 20 cells each; 3 technical replicates). Data show mean ± SEM. (**J**) The area of Poly-GA aggregates was lower in SRF-VP16 compared with SRF-VP16ΔMADS–expressing cells (*n* = 40 cells each). Data show mean ± SD. (**K**) In SRF-VP16–expressing cells, the Poly-GA/lysosome ratio was higher, suggesting more colocalization of aggregates in lysosomes compared with SRF-VP16ΔMADS (*n* = 40 cells each; 4 technical replicates). Data show mean ± SEM. Statistical testing was performed by 1-tailed *t* tests. Scale bar: 10 μm.

The aforementioned data suggest that SRF-VP16 induces autophagy-encoding genes in healthy cells, depicting SRF’s potential to regulate their gene transcription (Figure 7). Upon ALS-associated aggregate formation, however, SRF-VP16 had the reverse function and downregulated mRNA abundance of several autophagic genes induced by aggregates (Figure 7). Thus, SRF-VP16 could serve as a potentially novel tool to modulate autophagy and aggregate clearance in neurodegeneration. This potential SRF-VP16 function was analyzed by live recording of autophagy, Poly-GA aggregate clearance, and lysosomal colocalization (Figure 8).

Here, cells were transfected with a p62-GFP-mCherry construct, which indicates an enhanced autophagic flux by a green to red fluorescence switch (46). SRF-VP16^+^ (Figure 8, C and D, and Supplemental Video 2) but not as much SRF-VP16ΔMADS^+^ cells (Figure 8, A and B, and Supplemental Video 1) notably lowered the GFP/mCherry ratio (Figure 8I). This indicated more p62 presence in autophagy vesicles upon SRF-VP16 expression. Furthermore, the area of C9orf72-derived Poly-GA aggregates was measured (Figure 8, E–H). Here, SRF-VP16 (Figure 8, G and H, and Supplemental Video 4) reduced the aggregate size compared with SRF-VP16ΔMADS-expressing cells (Figure 8, E, F, and J, and Supplemental Video 3). Finally, the colocalization of aggregates with lysosomes was measured to estimate a potential clearance of Poly-GA aggregates in lysosomes (Figure 8, E–H, and K). In SRF-VP16^+^ cells, a more significant overlap of Poly-GA aggregates and lysosome signals (yellow in Figure 8, G and H) was observed compared with SRF-VP16ΔMADS (Figure 8, E and F). Overall, the Poly-GA/lysosome ratio was 0.8 for SRF-VP16 and approximately 0.3 for SRF-VP16ΔMADS, suggesting more Poly-GA clearance in lysosomes by SRF-VP16 (Figure 8K).

In summary, cell culture showed enhanced autophagic aggregate clearance by SRF-VP16.

### Neuronal activity modulates ALS-associated autophagy and disease burden through SRF.

Previous chemogenetic studies have demonstrated that increased neuronal excitability promotes neuroprotection in MNs by reducing the accumulation of misfolded SOD1. Conversely, decreasing MN excitability exacerbates the disease burden (18). To date, no TF has been identified to connect neuronal activity with such neuroprotective cellular processes, including reduction of misfolded SOD1 abundance and autophagy.

**Figure 9:**
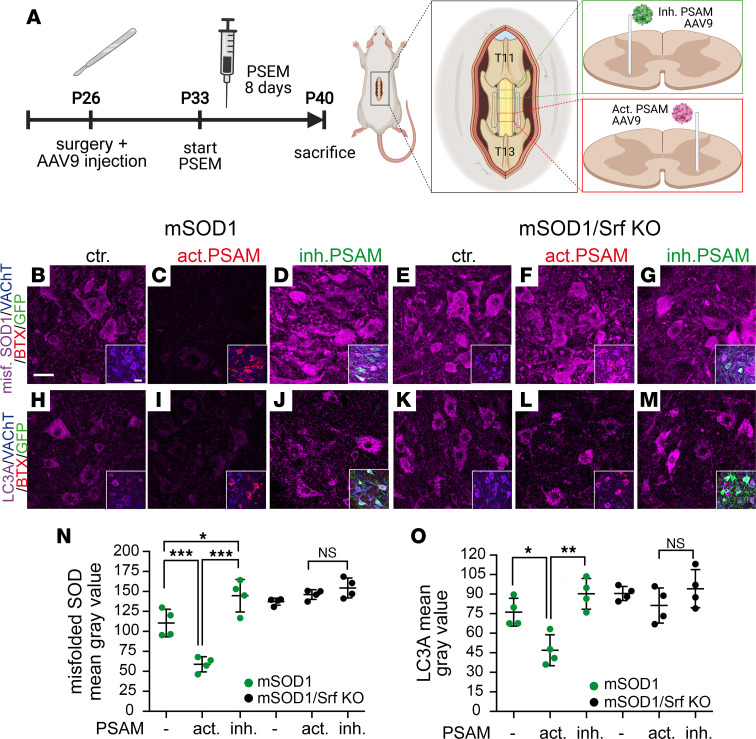
Chemogenetics reveals a requirement of SRF for neuronal activity to modulate disease burden in ALS-affected MNs. (**A**) P26 mice were injected into the spinal cord (level T11–T13) with AAV9 particles driving inhibitory (inh. PSAM labeled with GFP in green) or activatory PSAM expression (act. PSAM labeled with BTX in red). At P33, mice were injected for 8 days with the PSEM ligand. After sacrifice, ventral horns were stained for misfolded SOD1 and LC3A (along with VAChT, BTX, and GFP). Diagram created with BioRender.com with agreement no. ON244OBBZ4. (**B**–**G**) MNs of mSOD1 (**B**–**D**) or mSOD1/Srf-KO (**E**–**G**) mice were stained for misfolded SOD1 abundance. In mSOD1 mice, activating PSAM (**C**) lowered, whereas inhibitory PSAM (**D**) enhanced misfolded SOD1 level compared with mock infection (ctr.; **B**). In mSOD1/Srf-KO mice, chemogenetic manipulation failed to alter misfolded SOD1 levels (**E**–**G**). Inserts in **B**–**G** show a merged picture of all channels. (**H**–**M**) LC3A levels were decreased by activating PSAM (**I**) and slightly enhanced by inhibitory PSAM (**J**) compared with control (**H**) in mSOD1 mice. In SRF-deleted mice, neuronal excitability did not change LC3A abundance (**K**–**M**). Inserts in **H**–**M** show a merged picture. (**N** and **O**) Quantification of mean gray values for misfolded SOD1 (**N**) or LC3A (**O**). In **N** and **O**, *n* values are indicated by dots each reflecting 1 animal. In **N**, *n* values for MNs in mSOD1 mice were 150 (PSAM neg.), 198 (activating PSAM), and 200 (inhibitory PSAM) and, for mSOD1/Srf-KO mice, 148 (PSAM neg.), 150 (activating PSAM), and 200 (inhibitory PSAM). In **O**, *n* values for MNs in mSOD1 mice were 153 (PSAM neg.), 200 (activating PSAM), and 200 (inhibitory PSAM) and, for mSOD1/Srf-KO mice, 191 (PSAM neg.), 150 (activating PSAM), and 200 (inhibitory PSAM). Statistical testing was performed by 1-way ANOVA with Tukey corrections. Scale bar: 30 μm.

In the next step, we asked whether SRF could provide a transcriptional link between neuronal excitability and neuroprotective programs. For this, we employed pharmacologically selective actuator/effector module (PSAM/PSEM) chemogenetics (47) in mSOD1/SRF WT and mSOD1/Srf-KO mice. We hypothesized that chemogenetic modulation of neuronal excitability in diseased MNs should fail in the absence of SRF (Figure 9). To address this, the PSAM coupled either to 5HT3-receptor (PSAM-Act; positive MNs labeled with BTX in red) for neuronal depolarization or to a glycine-receptor (PSAM-Inh; positive MNs labeled with GFP in green) for neuronal hyperpolarization was overexpressed in MNs (Figure 9A). In all 3 conditions (ctr, PSAM-Act and PSAM-Inh), the expression levels of SRF in MNs was comparable (Supplemental Figure 9).

In line with previous reports in mSOD1 mice (18), enhanced neuronal activity (activating PSAM; Figure 9C) decreased, whereas lowering neuronal activity through inhibitory PSAM (Figure 9D) enhanced the misfolded SOD1 burden in MNs (Figure 9N) compared with the control (no AAV9 infection; Figure 9B). Besides misfolded SOD1 accumulation, autophagy was assessed through quantification of LC3A abundance in mSOD1 mice (Figure 9, H–J, and O). In alignment with previous reports (19, 48), here we showed that activating PSAM (Figure 9I) decreases, whereas inhibitory PSAM (Figure 9J) increases the autophagic response in ALS-diseased MNs (Figure 9O).

In the next step, chemogenetics was also performed in mSOD1/Srf-KO mice (Figure 9, E–G, and K–M). In contrast to the mSOD1 mice with WT SRF, chemogenetic-mediated elevation of neuronal excitability with activating PSAM failed to lower the misfolded SOD1 level in MNs (Figure 9F) compared with control (Figure 9, E and N). Likewise, inhibitory PSAM overexpression did not alter misfolded SOD1 levels compared with control (Figure 9, K, M, and N). As seen in misfolded SOD1, levels of LC3A were not altered in mSOD1 mice as a result of chemogenetic manipulation in the absence of SRF (Figure 9, K–M, and O).

This indicates that neuronal excitability engages SRF to exert neuroprotective processes in ALS diseased MNs.

## Discussion

**Figure 10:**
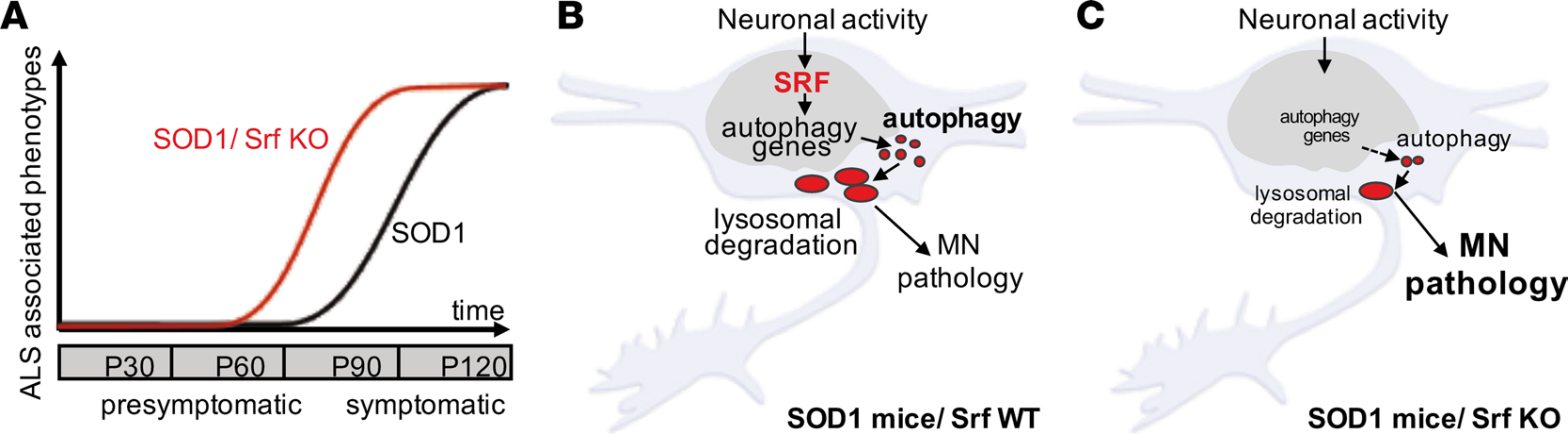
SRF connects neuronal activity–mediated gene transcription with autophagy in ALS MNs. (**A**) In mSOD1 mice with SRF (black line), ALS-associated phenotypes such as body weight loss and impaired motor function appeared at around P90. This disease onset was shifted to approximately P50 in mSOD1 mice with SRF deletion (mSOD1/Srf KO; red line). At the disease endpoint, both cohorts were similar. (**B**) In mSOD1 mice, neuronal activity activates SRF-mediated gene transcription resulting in autophagic gene induction in MNs. This might facilitate removal of inclusions through autophagy and lysosomes, thereby improving neuronal survival. (**C**) In mSOD1/Srf-KO mice, loss of SRF limits the induction of an autophagy program by neuronal activity, thereby precipitating MN vulnerability. This contributes to impaired MN function and, subsequently, a premature disease onset.

In the present study, we demonstrated that a prototypical activity-dependent TF, SRF, is required for the upregulation of autophagy genes in response to misfolded SOD1 accumulation in SOD1^G93A^ mice. MN-selective SRF deletion caused an earlier disease onset (Figures 2 and 3). Surprisingly, the effect of SRF deletion progressively disappeared. At around P90, no difference was observed in relation to performance, survival, or disease marker burden (Figure 2, Figure 3, and Figure 4). Furthermore, female mSOD1/Srf-KO mice showed less severe phenotypes compared with males, in line with previous reports (39). Overall, this suggests that SRF is critical for reducing FF-MN vulnerability at an early stage of disease progression (around P50–P60) but likely not for other MN subpopulations (Figure 10A). Additionally, this difference in early versus late SRF involvement might also correlate with an early hyperexcitabilty versus a later hypoexcitability of MNs during disease progression (see below). Importantly, SRF constitutes the first molecular link between neuronal activity and proteostatic autophagy responses in MNs, suggesting that the former may contribute to MN vulnerability by controlling the latter.

SRF, together with CREB, is a major TF in response to synaptic activity (23, 24). In fact, SRF-mediated transcriptional activation is a signature of physiological and pathophysiological states associated with increased synaptic input and neuronal activity. For propagation of physiological neuronal activity, SRF has been linked to the long-term remodeling of neuronal structure and function upon experience; loss of SRF interferes with the induction of both long-term potentiation (28) and long-term depression (27, 49). Similarly, in pathology, neuronal activity elicited by traumatic brain injury (36), Alzheimer’s disease (50, 51), or epilepsy (25, 26) target SRF. So far, SRF has mainly been associated with neuroprotective functions including axonal growth in development (52–55) and neuronal injury (32, 33, 36). The premature disease onset upon SRF loss in the SOD1 mouse ALS model is also congruent with such a neuroprotective SRF function in spinal MN neurodegeneration. In fact, SRF appears necessary in order to sustain FF-MNs, suggesting that FF-MNs may be more dependent on activity-regulated transcriptional programs. In agreement with this premise, SRF was strongly present in FF-MN (Figure 1), and a decrease in SRF^+^ neurons can be elucidated in terms of their high vulnerability to disruption of synaptic inputs and excitation.

Until now, one of SRF’s main transcriptional outputs was IEG regulation, including Fos and Egr family members ATF3 and Npas4. Together with SRF, these downstream TFs may propagate a further neuronal gene expression wave, thereby granting SRF a widespread footprint on the neuronal transcriptome. Interestingly, overexpression of the SRF transcriptional target ATF3 improves motor function in SOD1^G93A^ mice (56).

Herein, we observed an SRF role in transcriptional regulation of cellular proteostasis, which was previously unappreciated. The expression of 8 autophagy-encoding genes was affected by SRF deletion in mSOD1 mice (Figure 6). This resulted in an overall lower protein level (Figure 5) and mRNA abundance (Figure 6) of autophagic genes in SRF-deficient MNs. Conversely, SRF gain of function enhanced mRNA abundance of several autophagy-encoding genes and elevated autophagy, Poly-GA aggregate removal, and lysosomal degradation on the cellular level (Figures 7 and 8).

Overall, SRF gain and loss of function suggest a novel SRF role in the modulation of ALS-associated autophagy and proteostasis. In vulnerable MNs, such an impaired autophagy induction might contribute to the earlier disease onset observed upon SRF deletion (Figure 10). Thus, in WT mice, functional SRF provides a neuroprotective function to SOD1-affected MNs. In diseased MNs, SRF ensures a certain extent of autophagy- and lysosome-dependent aggregate clearance, thereby providing some neuroprotection to MNs (Figure 10B). Once SRF is depleted, autophagy and lysosomal degeneration is reduced and worsens MN pathology (Figure 10C). In alignment with this, we demonstrated that SRF-VP16 can decrease disease burden (Figure 8). Here, a C9orf72-dependent ALS assay was used in addition to the SOD1 mouse model, allowing for the analysis of C9orf72-derived protein aggregates. Thus, SRF activity affected neurodegeneration in 2 different ALS-associated mutant proteins, suggesting that SRF’s neuroprotective function is not limited to single ALS experimental models.

Bioinformatic analysis and ChIP revealed direct SRF binding sites in the promoter of 5 of 18 autophagy genes (Supplemental Table 2 and Supplemental Figure 6). This suggests that SRF may directly regulate some autophagy-encoding genes. However, this also implies that other TFs such as CREB- (57) or SRF-directed IEGs known to regulate autophagy (58, 59) might provide a link between neuronal activity and autophagy gene regulation. So far, only 2 instances of an SRF connection with autophagy have been reported (60, 61). Here, SRF protected MNs from rotenone-induced cellular death and reduced synuclein aggregation; however, this beneficial effect was abolished by either *Beclin1* or *Atg5* knockdown, implying that autophagy induction is a critical component of the neuroprotective SRF effects (60). This cell culture finding supports our conclusion that, in response to neuronal activity, SRF modulates autophagy to convey neuroprotection. Finally, SRF was shown to be subject to autophagy-dependent degradation (61). This points at a reciprocal regulatory mechanism whereby SRF might regulate mRNA abundance of autophagy-encoding genes. In turn, this may have a negative feedback on SRF abundance on the protein level.

As discussed above, SRF transcriptional regulation on autophagy-encoding genes appears as one mechanism by which SRF modulates disease progression in the SOD1 mouse model. However, given SRF’s wide impact on regulating a wealth of different target genes, including IEGs and actin cytoskeletal genes (23), those SRF-regulated gene classes might additionally contribute to the observed phenotypes.

In ALS pathogenesis, disruption of autophagic degradation of misfolded proteins is suggested to be a critical event (41–43, 62–64). Interestingly, 60% of vulnerable MMP9^+^ MNs were strongly positive for SRF (FF-MN; Figure 1). Thus, SRF loss might disproportionately affect this subpopulation, suggesting that sustaining a sufficient level of autophagy in FF-MNs may be an important protective mechanism and that activity-dependent transcriptional programs mediated by SRF may be involved in the autophagic response in FF-MN. It is worth noting that, in sporadic human ALS cases (Supplemental Table 1), MNs contained SRF structures reflecting nuclear rods (Supplemental Figure 1). These were not seen in mouse MNs, and this may be due to the specific SOD1^G93A^ mutation not producing these alterations. Although speculative, such rod-like nuclear structures in human MNs are reminiscent of cytoskeletal proteins (e.g., cofilin), which occur in nuclear rods of other neurodegenerative diseases (65, 66).

Is neuronal activity of FF-MNs necessary to uphold the autophagic pathway through SRF? The role of excitation and synaptic integrity in determining MN vulnerability in ALS is currently controversial. According to the excitotoxicity hypothesis, high levels of glutamatergic excitatory inputs — alone or together with increased intrinsic excitability of MNs — would be sufficient to drive MN cell death (67, 68). However, recent work has suggested that, rather than being hyperexcitable, MNs display an early increased excitability followed by hypoexcitability in vulnerable MNs later on (3, 13, 15, 69). This dynamic shift has also been observed in iPSC-derived MNs (17, 20, 70). Moreover, impaired synaptic function and reduced synapse numbers were shown in SOD1 mice and iPSC-derived MNs (19, 20, 71, 72). Thus, it is conceivable that the appearance of hypoexcitability and synapse loss may precipitate neuronal death by reducing SRF activation and terminally imbalancing the autophagic pathway. As previously demonstrated by chemogenetic control of MN firing (18, 19), one may predict that sustaining some degree of excitability may actually be beneficial. This prediction is corroborated in this study by showing that enhanced MN excitability not only lowers misfolded SOD1 levels (18) but also autophagy marker abundance (Figure 9). So far, no molecular mechanism has been identified as a target for neuronal activity’s neuroprotective impact on MNs. By combining the genetic deletion of SRF with chemogenetics in mice, this study revealed that SRF is required for neuronal activity to modulate disease progression (Figure 9). This provides a molecular framework with which to interpret the activity-dependent neuroprotection in ALS and possibly other neurodegenerative conditions as well (8).

In conclusion, this study provides a mechanistic link between neuronal activity, synaptic inputs, and autophagy imbalance (Figure 10). In doing so, we shed further light on a previously unappreciated role of SRF as a regulator of the neuronal proteostasis. These findings may have implications in other neurodegenerative conditions characterized by protein aggregates (such as Parkinson disease and Huntington disease) in which loss of neuronal firing appears to be the watershed event leading to the demise of vulnerable neuronal populations (8).

## Methods

### Transgenic mouse breeding strategy and housing

In order to get triple-transgenic *hSOD1^G93A^/ChAT^Cre^/Srf^loxp/loxp^* animals, *ChAT^Cre^* mice (The Jackson Laboratory, strain no. 006410) were first crossed with *Srf*-floxed mice (52, 73). It is worth noting that *ChAT^Cre^* mice allowed for the deletion of cholinergic cells such as MNs as well as other cell types, including skin cells (74). This mouse double-transgenic line (*ChAT^Cre^/Srf^loxp/loxp^*) was further crossed to high-copy hSOD1^G93A^ mice (The Jackson Laboratory, strain no. 002298) to get *hSOD1^G93A^/ChAT^Cre^/Srf^loxp/loxp^* triple-transgenic mice. This allowed for the motor neuron–specific *Srf* KO in an ALS mouse model. This triple-transgenic mouse line was maintained by mating *hSOD1^G93A^/ChAT^Cre^/Srf^loxp/wt^* males to *hSOD1^–^/ChAT^Cre^/Srf^loxp/wt^* females. From these breedings, 4 groups were derived: (a) WT (*hSOD1^–^/ChAT^Cre/^Srf^wt/wt^*), (b) mSOD1 (*hSOD1^G93A^/ChAT^Cre^/Srf^wt/wt^*); (c) Srf KO (*hSOD1^–^/ChAT^Cre^/Srf^loxp/loxp^*), and (d) mSOD1/Srf KO (*hSOD1^G93A^/ChAT^Cre^/Srf^loxp/loxp^*).

Mice were kept with free access to food and water in a pathogen-free animal facility at the University of Ulm, with a 12-hour day-night shift and appropriate temperature and humidity conditions. Humane endpoints of mSOD1 and mSOD1/Srf-KO mice were determined individually according to the criteria indicated below.

### Behavior tests and survival analysis

Mice were genotyped 21 days postnatally; they were then introduced blindly to a researcher. Behavior tests in the exploration and the in-depth cohort were carried out by different researchers during a similar day period (early afternoon). The researcher was introduced to the mice by handling and training at 3 weeks to reduce stress. From 4 until 20 weeks, all tests were carried out in the following order based on the stress level of the tests. In the pole test and inverted grid test, housing materials were applied at the bottom of the cage to prevent any possibility of injury from falling.

#### Body weight.

Body weight was measured twice a week, always before the other behavior tests. With a 0.1 g accuracy balance, the average of 3 measurements were recorded.

#### NS.

NS (37, 38, 40) was determined twice a week by tail suspension test, 25 cm walking test, and righting reflex. Briefly, holding 2 seconds for complete spreading of hindlimbs was considered NS = 0; partially collapsing and a normal gait was considered NS = 1; partially collapsing and slower walking with no more than 2 toe mistakes was considered NS = 2; total collapsing or more than 2 toe mistakes, but a righting reflex less than 10 seconds, was considered NS = 3; and at least 1 hindlimb paralysis or righting reflex over 10 seconds was considered NS = 4.

#### Pole test.

Pole tests were carried out once a week. Mice were put head up on the top of a vertical pole (rough wood, 50 cm high, diameter 1 cm). In healthy conditions, mice would make a turn and walk along the pole to reach the cage bottom. In disease conditions, mice would be slower, fail to turn, slide down the pole, or even fall from the pole. The time between starting to turn and reaching the bottom was regarded as the time to finish, while 2 minutes were recorded if mice failed or fell. Five tries were performed per mouse, and the best performance was recorded.

#### Inverted grid test.

The inverted grid test was performed once a week, during which a mouse was suspended from an inverted grid (barbed wire, 1 cm in between) to see how long it could hold itself. For a total of 3 minutes, the mice were given 3 tries, and the longest time was recorded (maximum of 3 minutes).

#### Grip strength.

Forelimb and fore-hind limb grip strength was measured twice a week using a grip strength meter at maximum strength mode (Panlab, Harvard Apparatus). The highest value within 3 tries was recorded.

#### Determination of survival and disease onset.

Health conditions, motor ability, and righting reflex were checked every day after mice reached NS = 3; meanwhile, water and wet food were provided on the cage bottom. A loss of 30% of the peak body weight or NS reaching 4 was considered a humane endpoint, while WT and Srf-KO mice were sacrificed at P200. After the endpoint, the individual body weight trend line was drawn, and disease onset was determined depending on the peak body weight age (38).

### Intraspinal injections of AAV

The following AAV vectors were intraspinally injected: activating PSAM pAAV (9)-pCAG-A7-floxed-PSAM (L141F, Y115F) 5HT3-WPRE and PSAM-Inhibitor pAAV (9)-pCAG-A7-floxed-PSAM (L141F, Y115F)-GlyR-GFP-WPRE. Intraspinal injection of AAV was performed as previously reported (18). Briefly, mice at day P26 were administered Buprenorphine (0.05 mg/kg; Reckitt Benckiser healthcare Ltd.) and Meloxicam (1.0 mg/kg; Metacam, Boehringer-Ingelheim) 30 minutes before isoflurane anesthesia (4% in O_2_ at 800 mL/min). Dorsal laminectomy was performed at the T11–T13 level, and the posterior median vein was used as reference to inject 2 sites. A pulled glass capillary coupled to a Picospritzer III apparatus was used to inject 0.5 μL AAV mixed with 0.5 μL 1.5% Fast-green (*y* = +0.25 mm, *z* = −0.4 mm). Different AAVs were injected 2 mm away (longitudinally), with both order and position being randomized. Once the injection was completed, muscles and skin were stitched with Prolene 7.0 surgical threads, and animals were transferred to single cages with facilitated access to food and water. Mice were monitored for 72 hours and were administered buprenorphine twice per day and meloxicam once per day. The PSEM308 effector molecule was administered from 7 days after surgery for 8 consecutive days at a dose of 5 mg/kg in saline. Mice were sacrificed 3 hours after the last agonist administration.

### Histology and immunofluorescence

Mice were anesthetized with 5 mg/kg ketamine and 5 mg/kg xylazine by i.p. injection and perfused with ice-cold PBS (2 mL/g), followed by 4% PFA in PBS (2.5 mL/g). Spinal cord lumbar enlargements were harvested and further fixed in 4% PFA in PBS at 4°C overnight, cryopreserved in 30% sucrose in PBS until sinking, embedded in OCT (TissueTek), and cross-sectioned on cryostat microtome at 40 μm (Leica 1900s). Free-floating fluorescence staining was performed as previously reported (37). In brief, spinal cord sections were blocked with 3% BSA and 0.3% Triton in PBS 2 hours, followed by primary antibodies (see antibody list in Supplemental Methods) in blocking solution on a shaker at 4°C for 48 hours. After washing (PBS, 3 × 30 minutes), secondary antibodies (see antibody list in Supplemental Methods) were incubated at room temperature for 2 hours. Samples went through washing steps before being mounted with ProLong Gold Antifade Reagent (Invitrogen). Similar procedures were performed for neuromuscular innervation analysis; however, gastrocnemius muscles were postfixed in 4% PFA for 2 hours and sectioned longitudinally at 30 μm; muscle samples were blocked with 6% BSA, 10% donkey serum, and 0.9% Triton in PBS.

### Confocal image acquisition and data analysis

All fluorescence images were acquired with LSM-710 confocal microscopy (Carl Zeiss) or a Leica DMi8 inverted microscope. Images were captured in 8-bit format at a 1,024 × 1,024 resolution and an optimal optical *z* resolution. Multichannel acquisitions were set up independently according to the excitation and emission spectra to get saturated and cross-talk free images. A *z*-stack with individual sections 1 μm apart was scanned continuously. Spinal cord images were acquired at the ventral horn. Gastrocnemius samples were acquired at L1 branch (1). All pictures were processed with ImageJ (FIJI; NIH), and a maximum intensity *z*-projection was performed. MN cell bodies were traced manually with a freehand selection tool recognized by VAChT or ChAT, followed by measurement of gray values of individual MNs. A threshold was set to avoid the background signal if a positive area was measured. MN numbers were counted in the original *z*-stack sequence. In the same staining, MNs from at least 5 ventral horns per mouse were analyzed. The NMJ innervation analysis was performed using the colocalization plugin, and an over 70% overlapping area of pre- and postsynapse was considered innervated. At least 100 L1-branch gastrocnemius NMJs per mouse were measured.

### Cell culture

HEK293 cells (from Leibniz-Institut DSMZ, Braunschweig, Germany) were transfected with polyethyleneimine and 2 μg of plasmid DNA in serum-free medium. After 5 hours, complete medium was added. Cells were transfected with the following constructs: GFP (as control), SRF-VP16, SRF-VP16 ΔMADS (44), constitutively active MRTF-A (52), Poly-GA (75), SOD1^G93A^ (47), SOD1^A4V^ (45), or P62-GFP-mCherry (46). For the Poly-GA–lysotracker experiment, after overnight incubation, 1 μM Lysotracker Deep Red (Thermo Fisher Scientific) was added and incubated for 2 hours at 37°C. For qPCR experiments with HEK293 cells, RNA was extracted with TRIzol (Qiagen). For reverse transcription, 1 μg RNA was mixed with random primers dN6 (Biomers), incubated for 10 minutes at 70°C, and placed on ice. Then, the master mix (room temperature 5× buffer, Promega Biosciences), dNTPs (Genaxxon), Ribolock RNase Inhibitor (Thermo Fisher Scientific), and M-MLV RT RNase (Promega Biosciences) were added. After 10 minutes at room temperature, the mixture was incubated at 42°C for 45 minutes, at 99°C for 3 minutes, and, thereafter, on ice.

### qPCR

qPCR was performed by mixing 2 μL cDNA, specific primer pairs and SYBR Premix Ex Taq (Tli RNase H Plus) PCR Master Mix (TaKaRa Bio Europe, Saint-Germain-en-Laye, France) in a total volume of 10 μL per well. The following settings were used in the Light Cycler 480 (Roche): 2 minutes at 50°C and 10 minutes at 95°C, followed by 50 cycles of PCR for 15 seconds at 95°C for denaturation and 1 minute at 60°C for annealing and elongation. The LC480 II software was used to detect the cycle threshold values. Relative mRNA expression of each target gene was calculated relative to the house-keeping gene *Gapdh* with the ΔCt method. All experiments were performed in technical duplicates. Primer details are provided in the Supplemental Methods.

### Live cell imaging

All live cell imaging was performed on the Nanolive 3D Explorer. Cells were imaged at 60***×*** magnification using the holotomography and fluorescence modalities. One frame was taken every 2 minutes for 1.5 hours. All frames were processed with ImageJ (FIJI), and a maximum-intensity *z*-projection was performed. A threshold was set to avoid background signal followed by mean gray value and area measurements.

### Statistics

All data were calculated in Excel (Microsoft Office 2019) and further compared in GraphPad Prism Software (v. 7.0). All values are expressed as mean ± SD unless otherwise indicated. The 1-tailed Student’s *t* test was applied to compare mean values in 2 groups. One-way ANOVA was applied for single time point markers among multiple groups. Paired 2-way ANOVA with Tukey correction was carried out to compare the multi–time point markers and behavior test results. Survival and onset analysis were performed using the Mantel-Cox statistical test. *P* < 0.05 was considered significant.

### Study approval

All animal experiments followed the institutional guidelines of the local animal facility (Tierforschungszentrum, Ulm University) and European laws. Animal experiments were approved by the Regierungspräsidium Tübingen (Tübingen, Germany). For human ALS samples, written informed consent was received prior to participation. The collection and the study were authorized by the ethics committee of Ulm University.

### Data availability

Data are available from the corresponding author upon request.

## Author contributions

JS and ND are co–first authors; JS is mentioned first since he started the project. BK and FR conceived the study and supervised the project. JS, ND, FR, and BK planned the experiments. DS and JS performed the behavior experiments. JS and ND performed the histological experiments, the imaging, and the image analysis. ND and JS performed the chemogenetic experiments. ND performed the cell culture, qPCR, and live cell imaging. JS, ND, FR, and BK prepared figures and drafted the manuscript. All authors read and approved the manuscript.

## Supplementary Material

Supplemental data

Supplemental video 1

Supplemental video 2

Supplemental video 3

Supplemental video 4

## Version 1 06/20/2023

In-Press Preview

## Version 2 08/08/2023

Electronic publication
