# Supplementary material for: SRF deletion results in earlier disease onset in a mouse model of amyotrophic lateral sclerosis
Source: JCI Insight. 2023 Aug 8;8(15):e167694. doi: 10.1172/jci.insight.167694 (PMC10445689; doi:10.1172/jci.insight.167694)
Supplement: Supplemental data [file jciinsight-8-167694-s141.pdf]

SRF deletion results in earlier disease onset in a mouse model of amyotrophic lateral sclerosis

Short title: SRF deletion accelerates ALS progression in the SOD1 mouse model

Jialei Song[1, 2, #], Natalie Dikwella[1, 2, #], Daniela Sinske[1], Francesco Roselli[2, 3]* and Bernd Knöll[1]*

[1]Institute of Neurobiochemistry
Ulm University
Albert-Einstein-Allee 11
89081 Ulm
Germany

[2] Department of Neurology
Ulm University
89081 Ulm
Germany

[3] German Center for Neurodegenerative Diseases (DZNE)-Ulm
89081 Ulm
Germany

Supplementary methods
Antibody list

Antibody	Dilution	Application	Source	Catalogue No.
α-Bungarotoxin Conjugated AF 555	1:500	IF	Invitrogen	B35451
α-Bungarotoxin Conjugated AF 647	1:500	IF	Invitrogen	B35450
Chicken anti-GFAP	1:500	IF	Abcam	ab4676
Goat anti-MMP9	1:500	IF	Sigma	M9570
Guinea pig anti-ChAT	1:300	IF	Synaptic Systems	297015
Guinea pig anti-VAChT	1:500	IF	Synaptic Systems	139105
Goat anti-VAChT	1:500	IF	Millipore	ABN100
Chicken anti-GFP	1:1000	IF	Abcam	ab13970
Rabbit anti-LC3A	1:300	IF	CST	4599S
Guinea pig anti-Synaptophysin1	1:300	IF	Synaptic Systems	101004
Mouse anti-β III tubulin	1:3000	IF	Eurogentec	MMS-435P-200
Mouse anti-misfolded SOD1(B8H10)	1:1000	IF	Medimabs	MM-0070
Mouse anti-P62	1:500	IF	Abcam	ab56416
Rabbit anti-Beclin 1	1:500	IF	CST	3738
Rabbit anti-Iba 1	1:500	IF	Wako	019-19741
Rat anti-LAMP1*	1:50	IF	DSHB	1D4B
Rat anti-SRF	1:50	IF	a kind gift from Prof. Dr. A. Nordheim, Tübingen University, Germany	
Rabbit anti-MRTF-A	1:500	IHC	Sigma	HPA030782
Rabbit anti-SRF	1:300	IHC	Santa Cruz	SC-335
Mouse anti-cFos	1:500	IF	Abcam	Ab208942
Mouse anti-CD68	1:200	IF	Abcam	Ab31630
Donkey anti-chicken CF 488	1:500	IF	Sigma	SAB4600031
Donkey anti-mouse AF 405	1:500	IF	Abcam	ab175658
Donkey anti-mouse AF 488	1:500	IF	Invitrogen	A21202
Donkey anti-goat AF 568	1:500	IF	Invitrogen	A11057
Donkey anti-guinea pig CF 405	1:500	IF	Abcam	SAB4600468
Donkey anti-guinea pig CF 568	1:500	IF	Biotium	20377
Donkey anti-guinea pig CF 633	1:500	IF	Biotium	20171
Donkey anti-rabbit AF 568	1:500	IF	Invitrogen	A10042
Donkey anti-rat AF 488	1:500	IF	Invitrogen	A21208
Donkey anti-mouse AF 647	1:500	IF	Invitrogen	A31571
Goat Anti-Rabbit IgG (H+L), Biotinylated	1:200	IHC	Vector	BA-1000-1.5

*The Rat anti-LAMP1 monoclonal antibody developed by Johns Hopkins School of Medicine, Pharmacology & Molecular Sciences, was obtained from the Developmental Studies Hybridoma Bank (DSHB), created by the NICHD of the NIH and maintained at The University of Iowa, Department of Biology, Iowa City, IA 52242.

Primer list

Mouse Gene	Primer Sequence
Gapdh	Fwd: 5´- TGG ATC TGA CGT GCC GC - 3' Rev: 5´- TGC CTG CTT CAC CAC CTT C - 3´
hSod1	Fwd: 5'- AGC ATG TTG AGC CGG CGA GT -3' Rev: 5'- AGG TTG TTC ACG TAG GCC GC -3'
Srf	Fwd: 5'- CAC GAC CTT CAG CAA GAG GA -3' Rev: 5'- CAA AGC CAG TGG CAC TCA TT -3'
cFos	Fwd: 5´- CCT GCC CCT TCT CAA CGA C - 3´ Rev: 5´- GCT CCA CGT TGC TGA TGC T - 3´
Egr1	Fwd: 5'- GCC GAG CGA ACA ACC CTA T -3' Rev: 5'- TCC ACC ATC GCC TTC TCA TT -3'
Npas4	Fwd: 5'- GCT ATA CTC AGA AGG TCC AGA AGG C -3' Rev: 5'- TCA GAG AAT GAG GGT AGC ACA GC – 3'
Atg101	Fwd: 5'- GAG CGA GCG GGT TCT CAT -3' Rev: 5'- TTC TCC ATT CCC AGC CAT CG -3'
Atg9a	Fwd: 5'- CTG CGG GTG ACA GTG AAT GA -3' Rev: 5'- ATT CCA GTT ACC AGC CTC GC -3'
Atg10	Fwd: 5'- GAG CGA GCG GGT TCT CAT -3' Rev: 5'- TTC TCC ATT CCC AGC CAT CG -3'
Atg14	Fwd: 5'- GCTGGAGTCTGTTCTGTGCT -3' Rev: 5'- TTGCTGTAGGCGGTAGTTGG -3'
Map1lc3a	Fwd: 5'- GTTCTGGTCAGGTTCTCCCC -3' Rev: 5'- TACCCAGGATGTGGTAGGCT -3'
Beclin1	Fwd: 5'- GGA AGA GGC TAA CTC AGG AGA GG -3' Rev: 5'- CTG TAG ACA TCA TCC TGG CTG GG -3'
Atg7	Fwd: 5'- CGG AAG TTG AGC GGC GA -3' Rev: 5'- CAG GAA AGC AGT GTG GAG TT -3'
p62	Fwd: 5'- GAATGTGGGGGAGAGTGTGG -3' Rev: 5'- TTCTGGGGTAGTGGGTGTCA -3'
Lamp5	Fwd: 5'- TCC TGC CTA CCT TCC TTC TCC -3' Rev: 5'- TTG CTA AAC CCT CCC ACC AG -3'
Ulk1	Fwd: 5'- CCC AAG CCA CCC TTT TCC TA -3' Rev: 5'- CAC ATC AGC TCC TTG TGG GG -3'
Fip200	Fwd: 5'- TGATTGAGTCTGTTAGTGCAGTC -3' Rev: 5'- ACACACAGTAATTCCACAGCAT -3'

Human Gene	Primer Sequence
GAPDH	Fwd: 5' - GAA GGT GAA GGT CGG AGT - 3' Rev: 5' - GAA GAT GGT GAT GGG ATT TC – 3'
VP16	Fwd: 5' - CTT AGA CGG CGA GCA CGT G – 3' Rev: 5' - CCC AAC ATG TCC AGA TCG AAA – 3'
GFP	Fwd: 5′ - GAAGCGCGATCACATGGT - 3′ Rev: 5' – CCATGCCGAGAGTGATCC - 3′
CFOS	Fwd: 5' - GGG GCA AGG TGG AAC AGT TA – 3' Rev: 5' - AGT TGG TCT GTC TCC GCT TG – 3'
ATG9A	Fwd: 5' – GGCAGGGTGTTCATTTTGG – 3' Rev: 5' – GTATCTCCCACACCAACGAC – 3'
ATG10	Fwd: 5' – GAGCGAGCGGTTCTCAT – 3' Rev: 5' – TTCTCCATTCCCAGCCATCG – 3'
MAP1LC3A	Fwd: 5' – AAGCCAGGTGCAAGGAGAAA – 3' Rev: 5' – GGAGGGTCAGTCAGCAACTC – 3'
BECLIN1	Fwd: 5' – GAGGTGAAGAGCATCGGGG – 3' Rev: 5' – CTCGTGTCCAGTTTCAGGGG – 3'
ATG7	Fwd: 5' – TGTGGTTGCCGGAAGTTGA – 3' Rev: 5'- CCCAACATCCAAGGCACTACT – 3'
P62	Fwd: 5' – AGCGTCAGGAAGGTGCCATT – 3' Rev: 5' – CCCCATGTTGCACGCCAA – 3'
LAMP5	Fwd: 5' – ATTTGTGGTGCGGGAAAATGG – 3' Rev: 5' – CATGTTGTGGCTTTCATCTACG – 3'
ATG14	Fwd: 5' – TGGACTCCGTGGACGATGC – 3' Rev: 5' – CGCTCTGAACGCATTTGGC – 3'
ATG101	Fwd: 5' – GGGAGAGTGGTGGCATCTGA – 3' Rev: 5' – GGCACACGGTTAAAACAGCTC – 3'
ATG5	Fwd: 5' – ACCTTCTGCACTGTCCATCT – 3' Rev: 5' – CAATCCCATCCAGAGTTGCT – 3'

Immunohistochemistry (IHC) of ALS patient samples

Lumbar spinal cords from stage 1-2 ALS patients (TDP-43 pathology stages (75)) were fixed with formalin, embedded in paraffin and cross-sectioned at 50 µm and followed by a free-floating IHC. In brief, sections were washed in xylene to remove paraffin and followed by rehydration with alcohol set. Endogenous peroxidase was inactivated by 3% H_2O_2, then heat induced antigen retrieval with citrate buffer (pH 6.0) was performed. Samples were blocked in 5% BSA, 0.25% Triton-X and 18.2% DL-lysine, then incubated with 1st antibodies overnight (see supplement), biotinylated 2nd antibodies 2h, ABC-kit (Vector Laboratory) 1.5h and followed by a DAB chromogen reaction. Sections went through alcohol series and Histo-Clear (National Diagnostics) before being mounted. Pictures were acquired at the ventral horns on a brightfield microscope (Keyence BZ-X810) using Z-stack mode and followed by a full-focus using Analyzer software (Keyence).

Laser capture microdissection (LCM) and cDNA synthesis

For LCM, mice were anesthetized with 1 mg/kg body-weight ketamine chlorhydrate and 0.5 mg/kg xylazine. Once deep level anesthesia was confirmed by lack of a toe-pinch response, the chest cavity was exposed, the right atrium was nicked with sharp forceps and the left ventricle was infused with ice-cold PBS at a rate of 5-7 ml/min for 2 minutes with a peristaltic pump. Spinal cord samples were quickly dissected, embedded in OCT (TissueTek) and stored at -80°C. The embedded blocks were cryosectioned at -20°C to produce 12 µm slices on RNase-free polyethylene terephthalate (PET) membrane slides. Sections were fixed in 70% ethanol diluted in DEPC-H2O at -20°C and stained with 1% cresyl violet in 50% ethanol/DEPC-H2O for

1 minute each. Once stained, slides were incubated for 1 minute in 70% and 100% ethanol at +4°C. Then, 20 MNs were captured using the Laser Microdissection System (Palm MicroBeam, Zeiss) in a 500 µl clear adhesive cap (Carl Zeiss).

Cells were lysed by adding 21 µl 1x SuperScript III first-strand RT buffer (Invitrogen) containing 1% NP40 on the cap surface at 42°C for 20 min. The lysate was spun down and reverse transcription was performed by using the SuperScript III First-Strand Synthesis System of the RT-PCR kit (Invitrogen). Briefly, RNA was mixed with 50 ng/µl random hexamers and 10 mM dNTP mix, incubated at 65°C for 5 min and placed on ice for at least 1 min. Then, cDNA synthesis mix (10X RT buffer, 25mM MgCl2, 0.1M DTT, RNaseOUT 40 U/µl, SuperScriptIII RT 200 U/µl) was added and incubated 10 minutes at 25°C followed by 50 minutes at 50°C. The reaction was terminated at 85°C for 5 minutes. To remove excess RNA, RNase H was added to the solution and incubated for 20 minutes at 37°C.

Promoter analysis

Bioinformatic analysis was performed using Eukaryotic Promoter Database (EPD, https://epd.epfl.ch//index.php) to find potential SRF-transcription binding sites in autophagy related genes. In brief, autophagy related genes were screened in *Mus musculus* looking for SRF as motif, Transcription Factor Motifs (JASPAR CORE 2018 Vertebrates) as library, from -1500 to 100 bp relative to transcription start site (TSS), and a cut-off p-value of 0.001 was applied. After screening, DNA sequence at the binding site was checked manually to confirm the classical CArG box sequence $CC(A/T)_6GG$.

Chromatin immunoprecipitation

ChIP was performed as previously described (7). Primer sequences were as follows:

Egr1: Fwd: 5’ CCC ACC ACT CTT GGA TGG GAG GGC TTC AC 3’
Rev: 5’ TCG GCC TCT ATT TCA AGG GTC TGG AAC AGC 3’

Atg7: Fwd: 5' CCA CGC CCA GCC CAA ATG CT 3’
Rev: 5' ACC TTT GCT TTG CCC CAG TGC T 3'

Atg9a: Fwd: 5' GGC CAG CCC TGT ATA AAC AAC CC 3’
Rev: 5' GGT ATC CGG GTT GCT TTG GGG T 3’

Atg10: Fwd: 5' TGG CCC TAC CAT TGC CTC TTG GT 3’
Rev: 5' CGC GAG CAT CCC CAA GAG CCT A 3'

Supp. Table 1
Human ALS cases used

case	f/m	age	dur	so	pyr	NFT	Aβ	α-syn	ALS	MN
1	m	60	2.0	C	1	0	na	0	2	1
2	m	75	1.2	B	1	III	0	0	4	1
3	m	69	1.9	L	0	I	0	0	1	0
4	f	67	1.1	C	1	III	0	0	4	1
5	f	64	2.3	C	1	III	0	0	2	1
6	f	42	2.3	L	1	0	0	0	2	1

Abbreviations: f/m – female/male; **age** – age at death in years; **dur** – disease duration in years; **so** – **site of onset**: C – cervical (upper extremity), L – lumbar (lower extremity), B – bulbar; **na** – not available or not assessed; **pyr** – absence (0) or presence (1) of pyramidal signs; **MN** – involvement of spinal cord anterior horn α-motoneurons layer 9, all four levels (0 = no, 1 = yes); **NFT** – Alzheimer disease-related neurofibrillary tangle stage 0-VI using AT8 immunohistochemistry (IHC) [1]; **Aβ** – amyloid-β deposition phase 0-5 using Campbell-Switzer silver staining [2, 3]; **α-syn** – Parkinson disease-related neuropathological stage 0-6 using α-synuclein IHC [4]; **ALS** – neuropathological stages 1-4 based on regional distribution pattern of phosphorylated TDP-43 (pTDP-43) aggregates using IHC [5, 6].

Supp. Table 2
SRF binding sites in autophagy encoding genes

Gene name	CArG box position (bp before TSS)	Gene name	CArG box position (bp before TSS)
Atg5	1459	*Atg12*	none
Atg7	538	*Atg13*	none
Atg9a	835	*Atg 101*	none
Atg10	378	*Beclin1*	none
Map1lc3a	1522	*Lamp1*	none
Atg3	none	*Lamp2*	none
Atg4	none	*Lamp3*	none
Atg14	none	*Sqstm1 (p62)*	none
Atg16	none	*Ulk1*	none

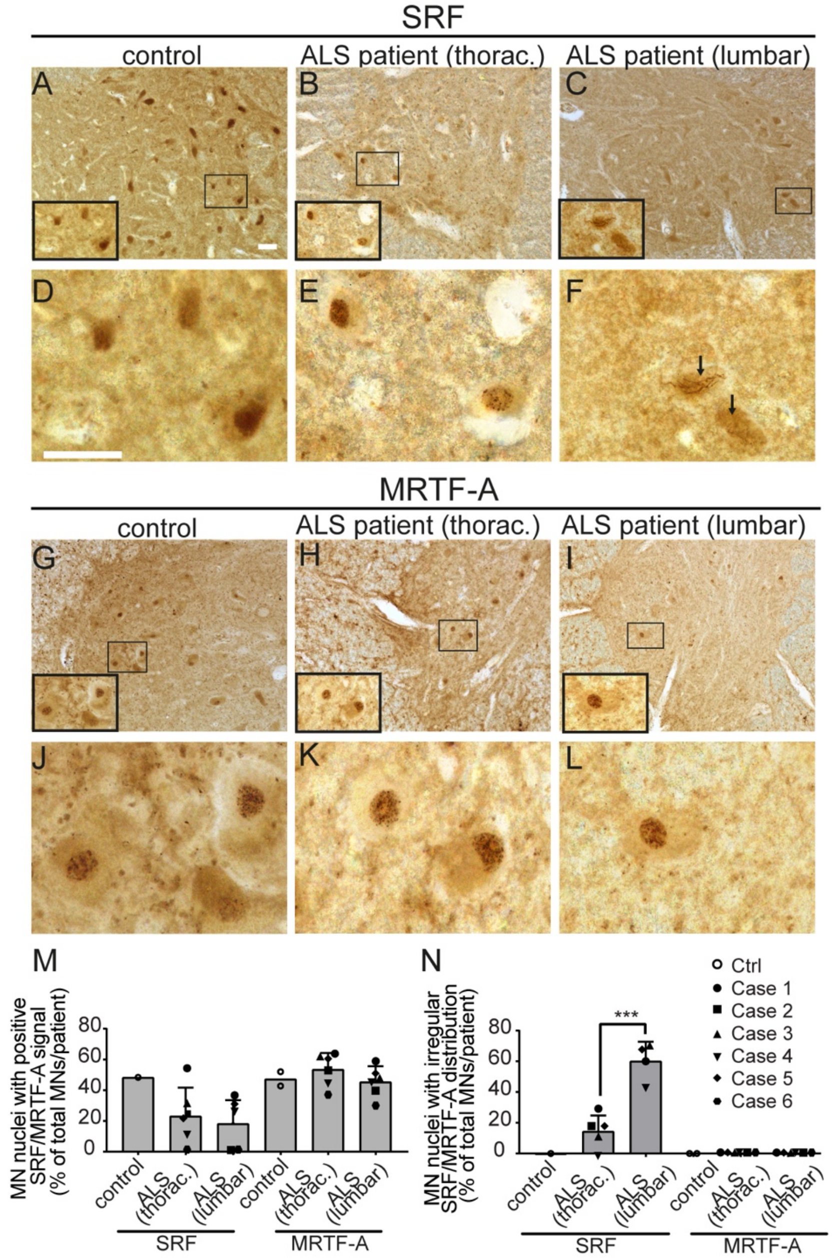

Supp. Fig. 1

SRF and MRTF-A are present in MNs of ALS patients

(A-F) Ventral horns from control (A, D) or ALS patients (B, C, E, F) were stained for SRF abundance. In MNs of a control patient SRF was uniformly localized in MN nuclei (A, D). In thoracical MNs (B, E), SRF was predominantly localized uniformly in nuclei with some nuclei presenting a speckled SRF localization (E). In lumbar MNs (C, F), SRF was restricted to the nucleus. However, SRF abundance was not uniform in several MNs and rod-shaped structures positive for SRF were observed instead (arrows in F).

(D-F) are higher magnifications of inserts presented in (A-C).

(G-L) MRTF-A was restricted to the nuclei of MNs of control (G, J) and in thoracical (H, K) or lumbar (I, L) MNs of ALS patients. In all cases, nuclear MRTF-A expression was found in a dotted pattern (see J, K, L).

(M) Quantification of SRF or MRTF-A positive MNs/patients in relation to the total MN number.

(N) Quantification of SRF or MRTF-A expression in nuclei appearing with an irregular shape (see arrows in F). Mainly for SRF in lumbar MNs an irregular expression pattern was observed. In (M, N) each dot reflects one patient analyzed. N numbers (SRF: ctrl, 118 MNs; ALS thorac., 201 MNs; ALS lumb., 218 MNs; MRTF-A: ctrl, 184 MNs; ALS thorac., 322 MNs; ALS lumb., 385 MNs). Statistical testing was performed by one way ANOVA with Tuckey corrections. Symbols in (M, N) indicate patients (see also Supp. Table 1).

Scale-bar (A-C; G-I) = 50 μm; (D-F; J-L) = 25 μm

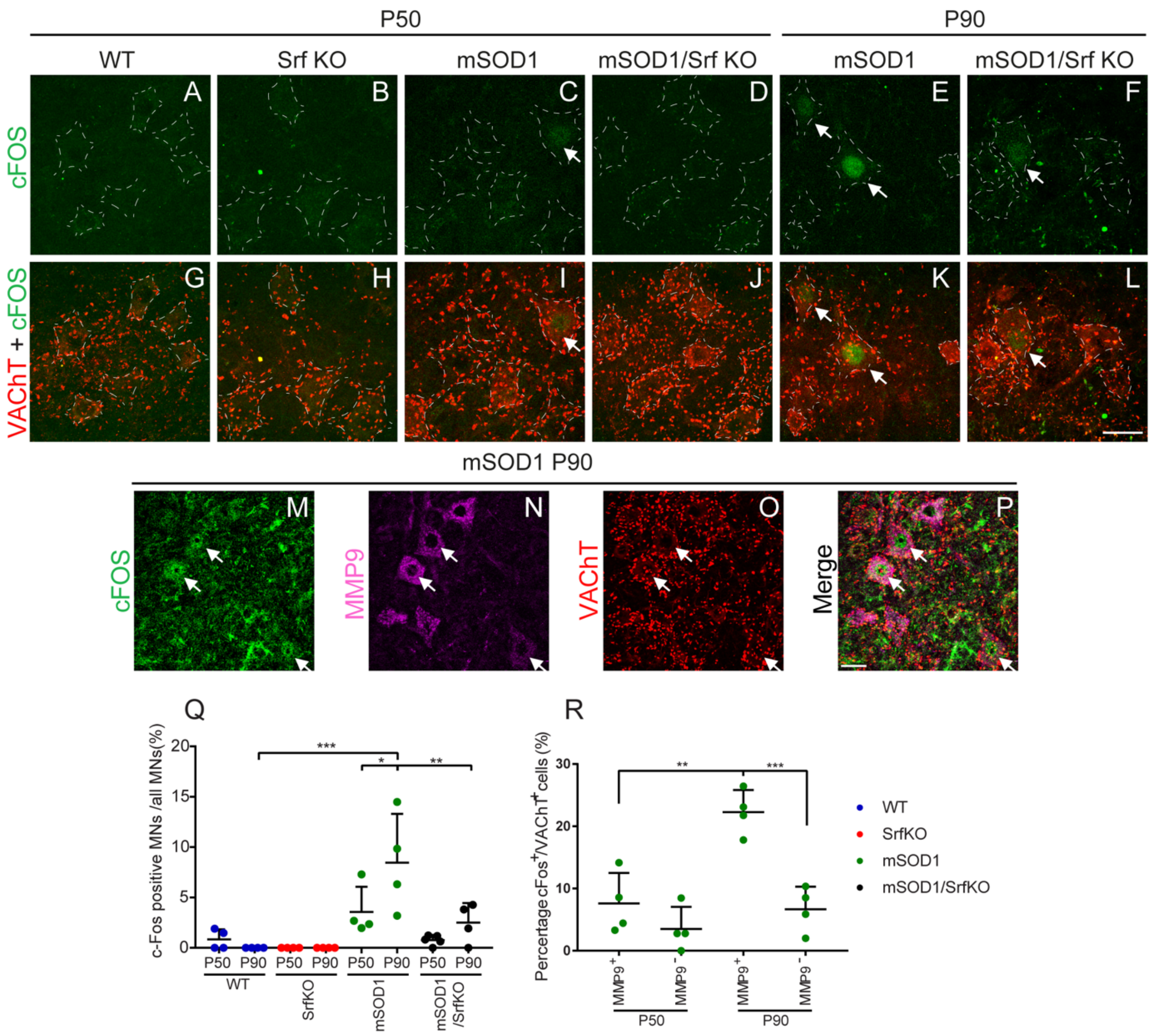

Supp. Fig. 2

c-Fos is upregulated in mSOD1 MNs at later stage but not in mSOD1/SrfKO.

A-L: Immunofluorescence staining of cFos (green) and VAChT (red) in ventral horns of the lumbar spinal cord. cFos positive neurons are labelled with arrows. C-Fos is absent in WT (A), SrfKO (B) and mSOD1/SrfKO (D) mice at P50. In contrast, a weak cFos signal is detected in ~4% of VAChT+ cells in mSOD1 at P50 (C).c-Fos is detected in ~10% VAChT+ cells with some strongly positive cells in mSOD1 mice at P90 (E) whereas only a weak c-Fos signal is detected in ~3% of VAChT+ cells in mSOD1/SrfKO at P90 (F). This was significantly lower as quantified in Q.

G-L: VAChT signals merged with c-Fos in A-F.

M-P: Immunofluorescence staining of c-Fos (green), MMP9 (magenta) and VAChT (red) in lumbar cord ventral horns. Upregulation of motoneuronal c-Fos signal is mostly limited to MMP9+ cells at P90.

Q: Quantification of cFos positive percentage in ventral horn VAChT+ cells.

R: Quantification of cFos positive percentage in mSOD1 ventral horn VAChT+ cells at P90, pooled by MMP9 signal.

Q, R: Each dot reflects one mouse. Four mice each condition were quantified.

Statistical test: one-way ANOVA.

Arrows: c-Fos positive MNs.

Scale bar: 30 μm.

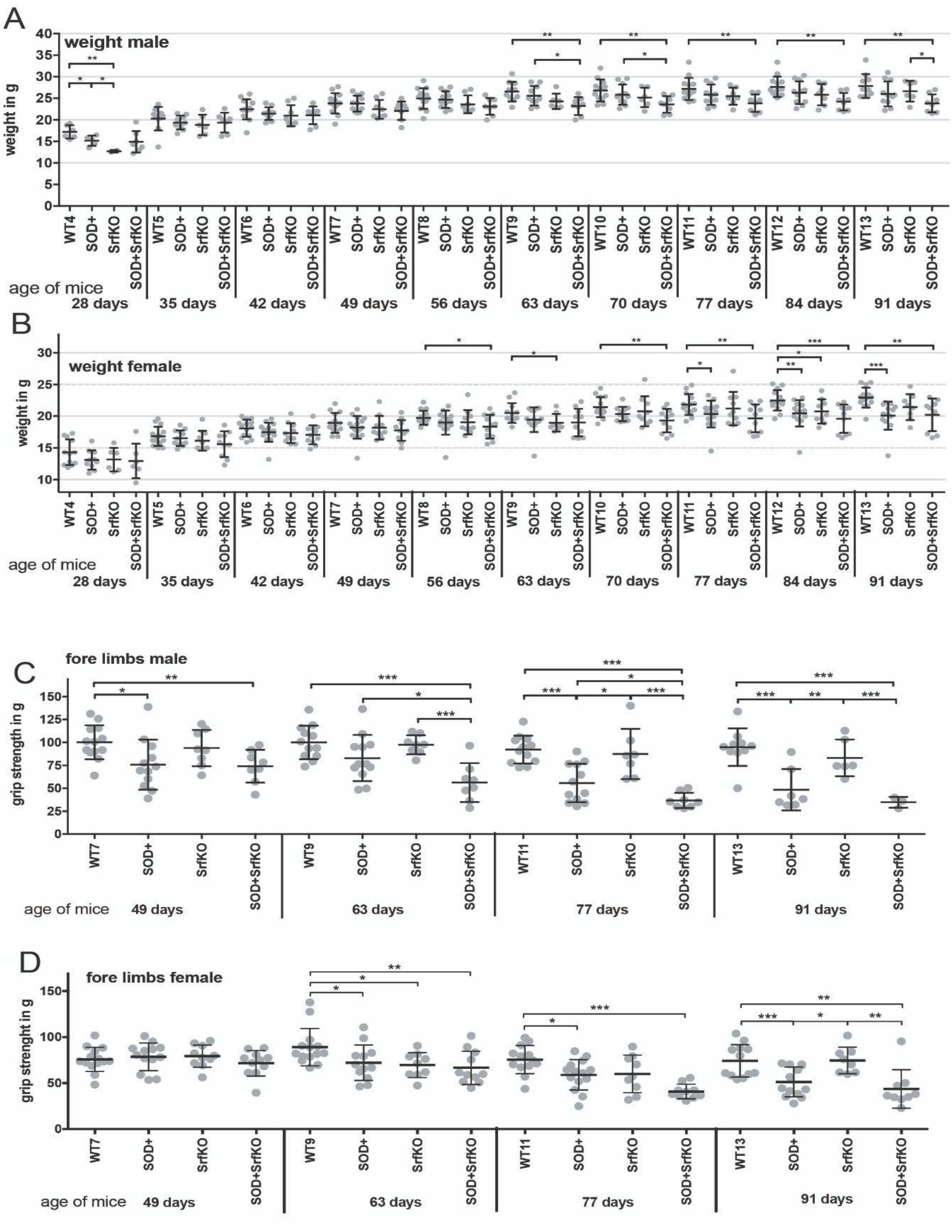

Supp. Fig. 3

Behavior analysis in the exploratory cohort

(A, B) Body weight analysis of all four cohorts (WR, Srf KO, SOD1, SOD1/Srf KO) starting at 28 days after birth until 91 days. In males (A) and less pronounced in females (B) SOD1/Srf KO animals have less weight compared to their SOD1 littermates.

(C, D) The forelimb strength was tested in male (C) and female (D) animals in all four cohorts. In SOD1 animals, forelimb strength decreased compared to WT and Srf KO animals. Grip strength was further decreased in SOD1/Srf KO animal compared to SOD1 animals at several timepoints. Statistical testing was performed by one way ANOVA with Tuckey corrections.

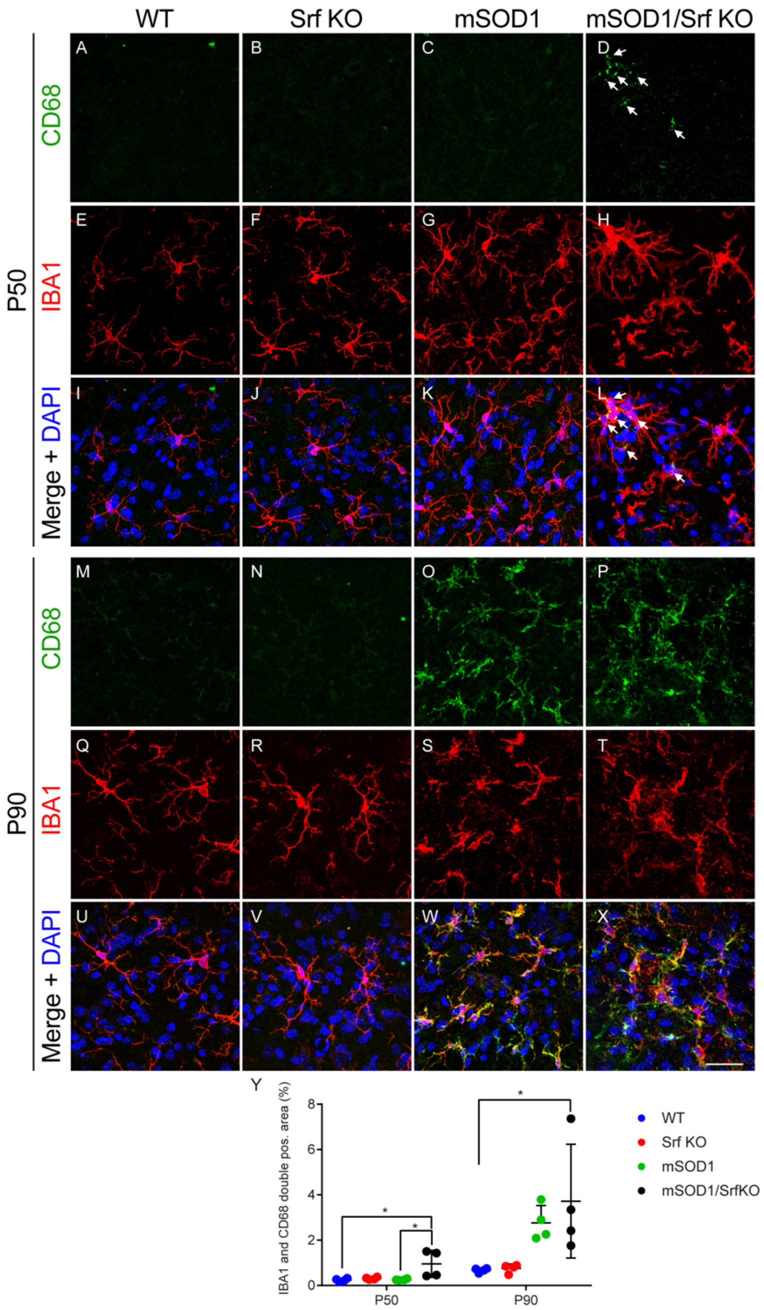

Supp. Fig. 4

Motoneuronal SRF deletion leads to an upregulation of CD68 positive microglia in mSOD1 lumbar cord at P50, but not at P90.

(A-X) Immunofluorescence staining of CD68 (green), IBA1 (red) and DAPI (blue) in all four genotypes at P50 (A-L) and P90 (M-X).

(A-C) CD68 shows very weak signal in WT, SrfKO and mSOD1 lumbar ventral horn at P50.

(D) CD68 is present in some IBA1 positive cells (arrows) in mSOD1/SrfKO at P50.

(E-H) IBA1 staining shows morphology of resident microglia in WT, SrfKO and mSOD1, but an activated morphology in mSOD1/SrfKO at P50.

(I-L) Merge of A-H.

(M-P) CD68 shows weak signals in WT (M) and SrfKO (N) at P90. A clear induction of CD68 positive cells is observed in mSOD1 (O) and mSOD1/SrfKO (P).

(Q-T) IBA1 staining shows morphology of resident microglia in WT and SrfKO, but an activated morphology in both mSOD1 and mSOD1/SrfKO at P90.

(U-X) Merge of M-T.

(Y) Quantification of CD68 IBA1 double positive area in spinal ventral horn.

Each colored dot reflects one mouse. 4 mice are recruited in each condition. Statistical test: one-way ANOVA. Arrows: CD68 IBA1 double positive cells. Scale bar: 30 µm.

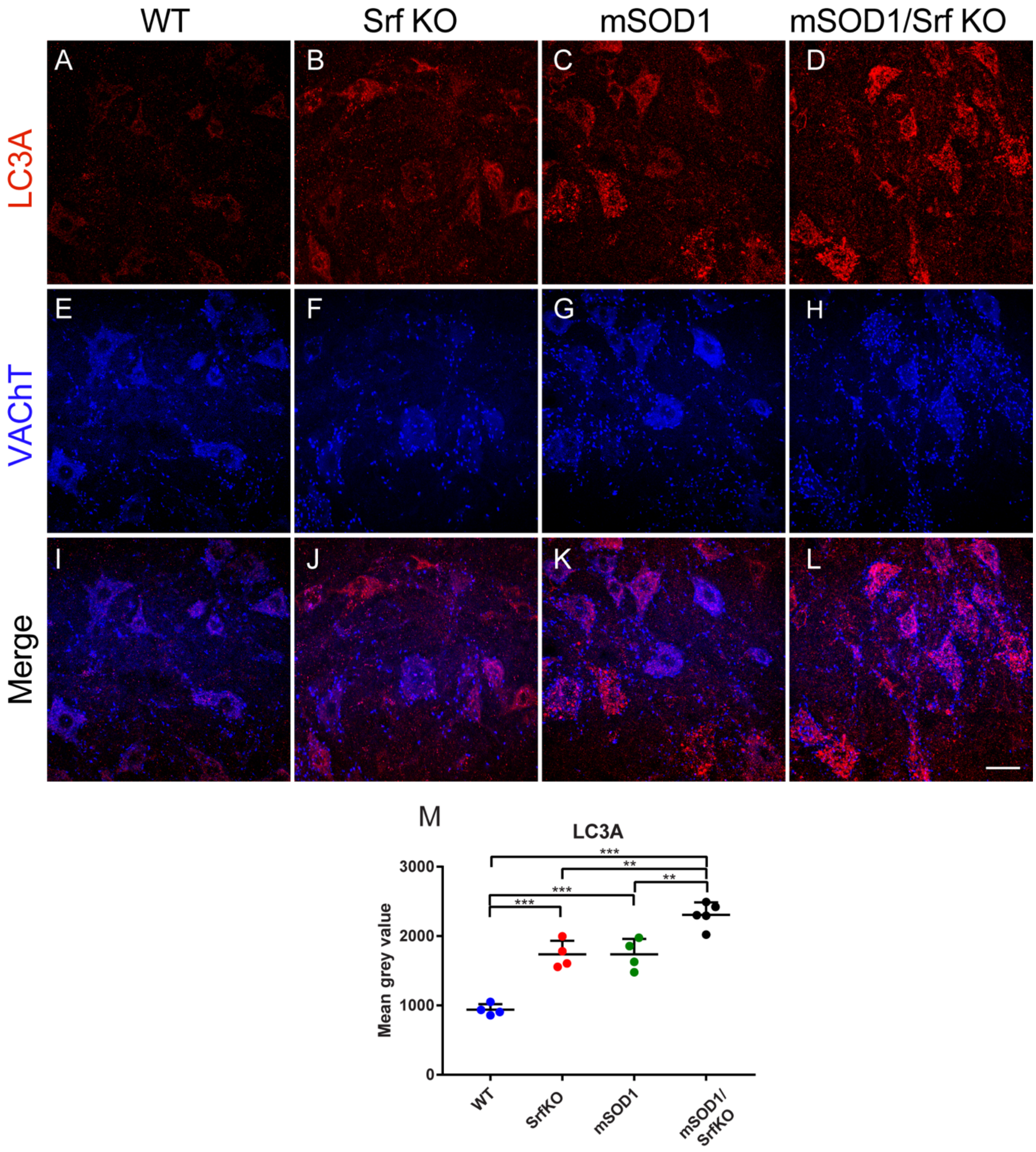

Supp. Fig. 5

Abundance of the autophagy marker LC3 is altered in mSOD1/SrfKO mice

(A-L) Immunofluorescence staining of LC3A (red) and VAChT (blue) in lumbar ventral horns at P50.

(A) In WT mice, LC3A showed a uniform weak signal in VAChT$^+$ cells.

(B) LC3A intensity was upregulated in VAChT$^+$ cells in SrfKO.

(C) LC3A formed high intensity puncta in VAChT$^+$ cells in mSOD1 mice.

(D) LC3A puncta abundance was further upregulated and filled in cytoplasm of VAChT$^+$ cells in mSOD1/SrfKO mice

(M): Average intensity quantification of LC3A. Each colored dot represented an individual mouse. Significance was tested with one-way ANOVA. Scale bar (A-L): 30 μm

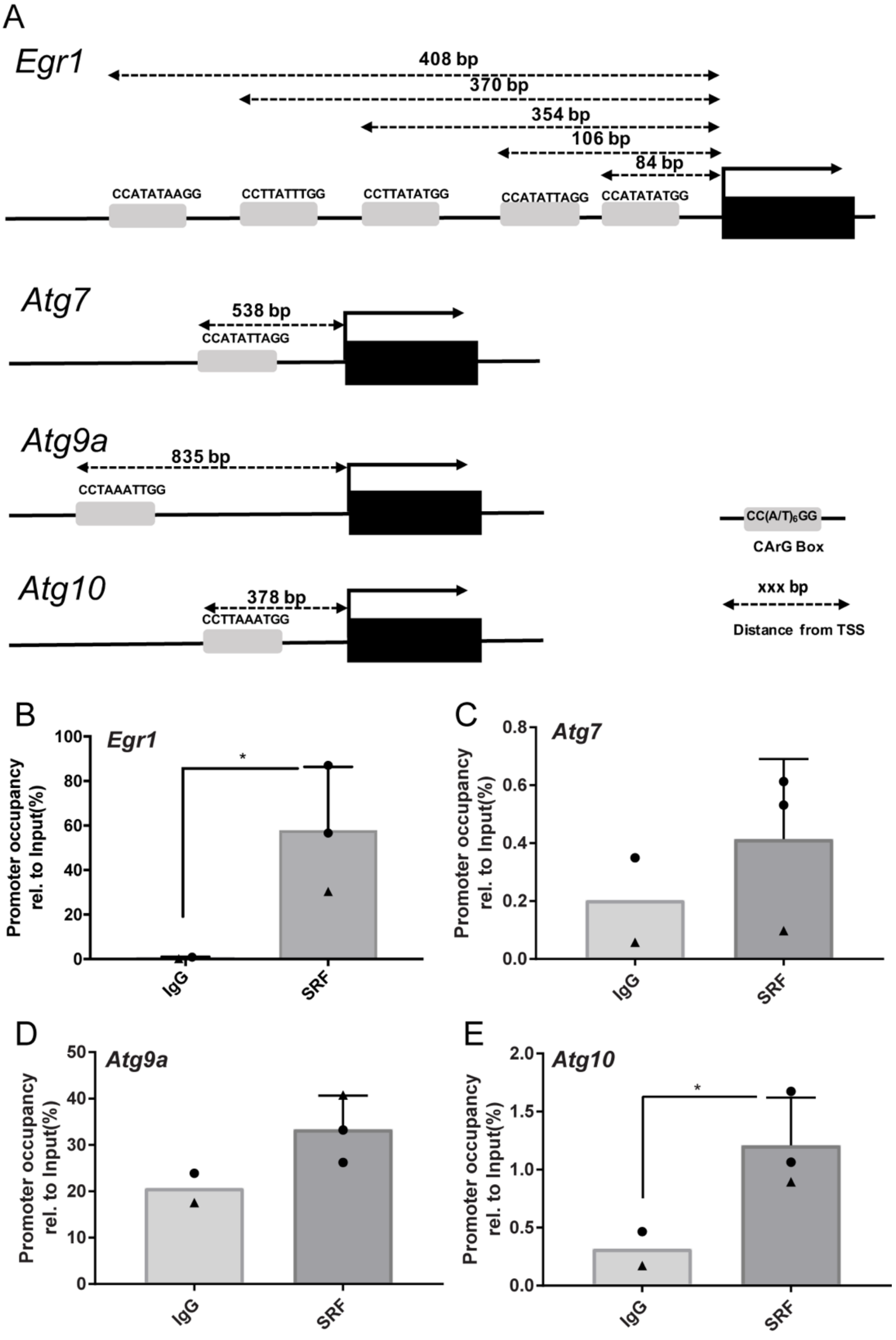

Supp. Fig. 6

Chromatin immunoprecipition (ChIP) shows SRF promoter binding to some autophagy encoding genes

(A) Analysis for SRF binding sites, so-called CArG boxes, in the promoter regions of autophagy encoding genes. *Egr1*, a prototype SRF regulated gene contains five CArG boxes within approx. 400 bp before the transcriptional start site and served as positive control. Three autophagy encoding genes, *Atg7*, *Atg9a* and *Atg10*, also have single consensus CArG boxes in close proximity to the transcriptional start site.

(B-E) ChIP analysis in NIH 3T3 cell lysates incubated with anti IgG (control) or anti SRF directed antibodies. *Egr1* (B) showed strongest SRF binding but also *Atg7* (C), *Atg9* (D) and *Atg10* (E) promoters were bound by SRF. Each dot reflects one independent experiment.
Statistical test: T Test.

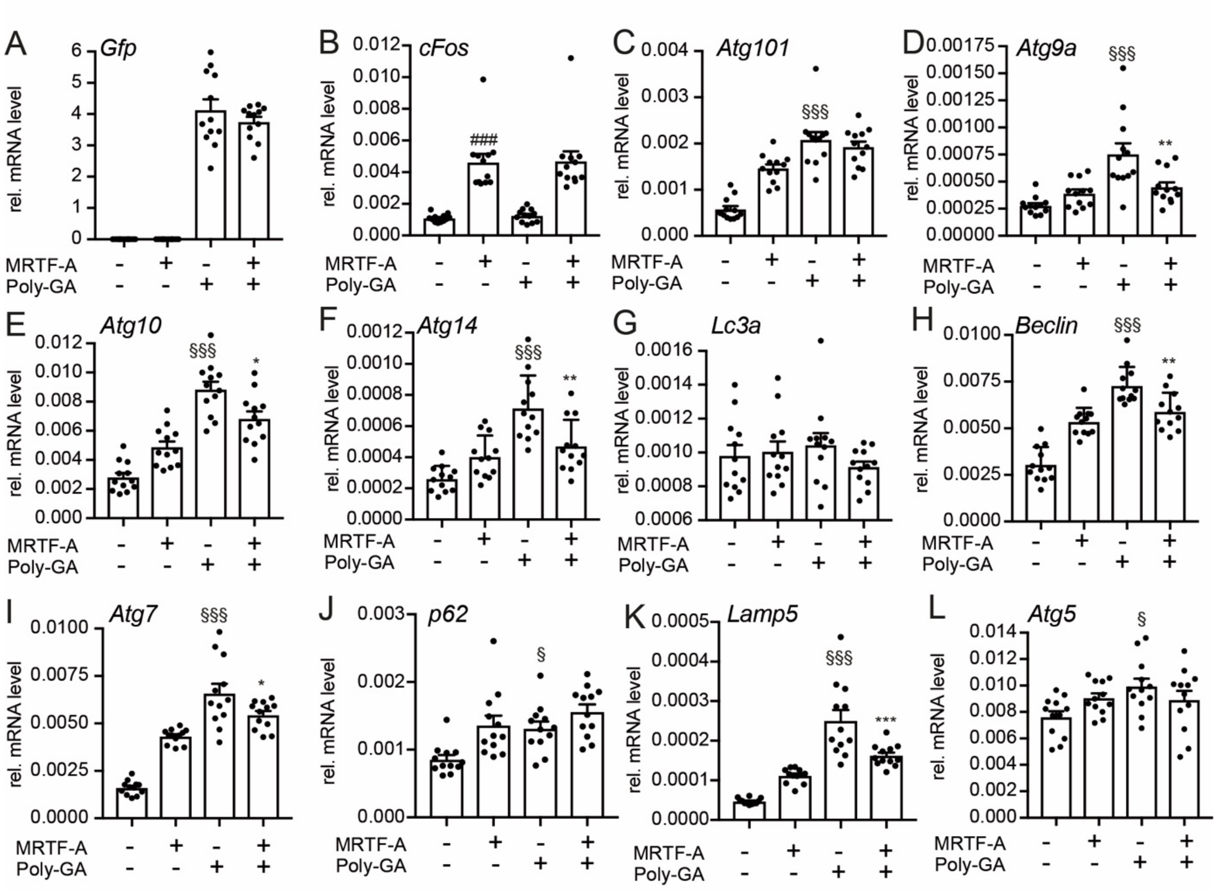

Supp. Fig. 7

Constitutively-active MRTF-A modulates mRNA abundance of autophagy associated genes

Hek293 cells were either mock transfected or express constitutively-active MRTF-A the presence or absence of aggregates formed by poly-GA expression. Subsequently, qPCR was performed to assess mRNA abundance of genes indicated.

(A-B) Expression of GFP tagged poly-GA (A) was similar on mRNA level. MRTF-A induced *cFos* abundance (B).

(C-L) MRTF-A induced *Atg101* (C), *Atg10* (E), *Atg14* (F), *Beclin1* (H), *Atg7* (I), *p62* (J) as well as *Lamp5* (K). Poly (GA) expression upregulated *Atg101* (C)*, Atg9a* (D), *Atg10* (E), *Atg14* (F), *Beclin1* (H), *Atg7* (I), *p62* (J) and *Lamp5* (L). MRTF-A down-regulated those autophagy (*Atg9a, Atg10, Atg14, Beclin1, Atg7*) and lysosome (L*amp5*) encoding genes induced by poly (GA) aggregate formation.

In (A-L) each black dot reflects one cell culture dish.

*, #, § denote significance between MRTF-A and MRTF-A in the presence of Poly-GA (3rd vs. 4th bar), between mock and MRTF-A (1st vs. 2nd bar) and between mock and poly GA (1st and 3rd bar), respectively. Statistical testing was performed by one way ANOVA with Tuckey corrections.

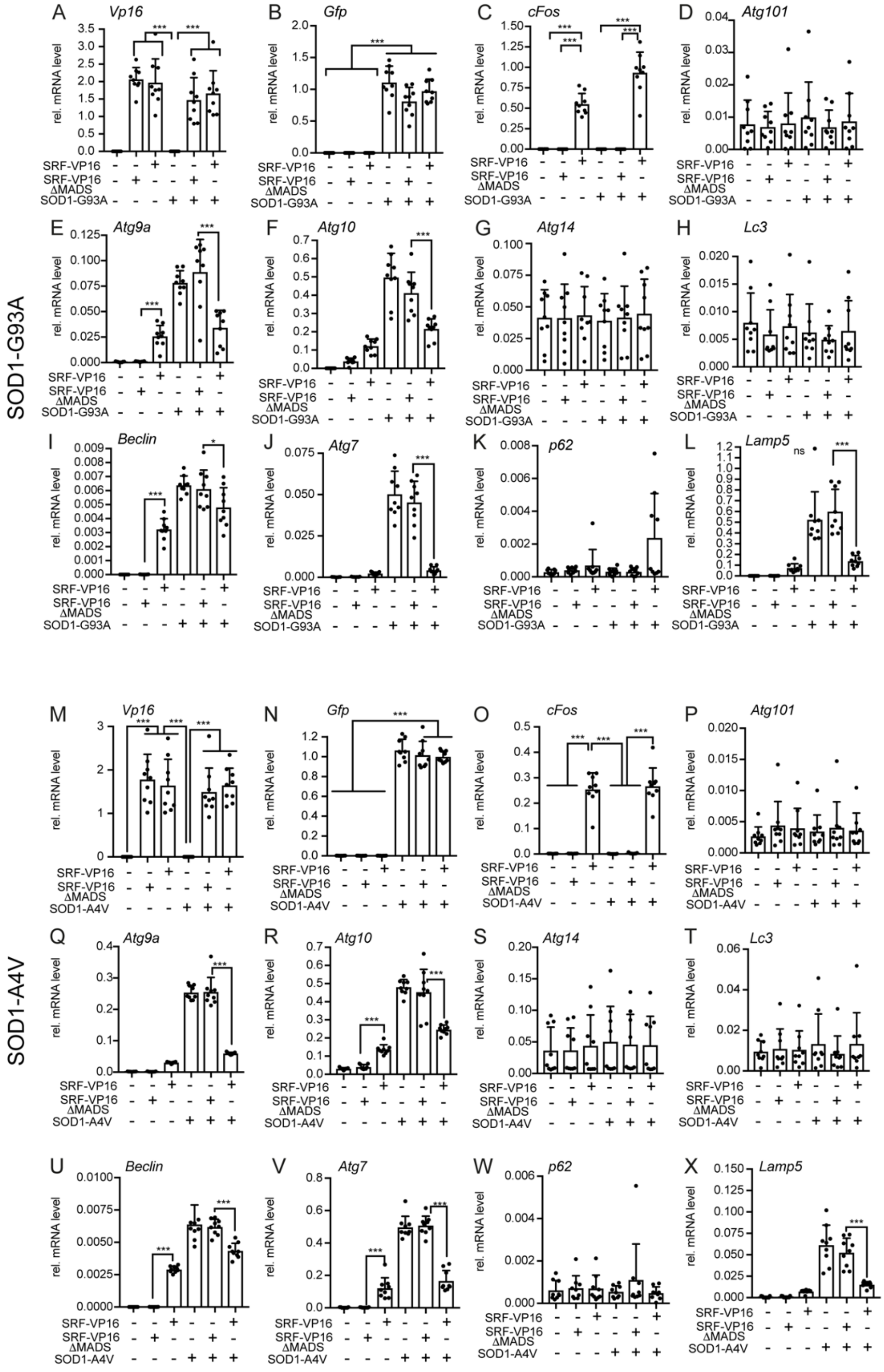

Supp. Fig. 8
SRF-VP16 reduces SOD1associated induction of autophagy genes

HEK293 cells expressed either constitutively-active SRF-VP16 or inactive SRF-VP16ΔMADS in the presence or absence of aggregates formed by either SOD1^{G93A} (A-L) or SOD^{1A4V} (M-X). Subsequently, qPCR was performed to assess mRNA abundance of genes indicated. SRF-VP16 but not SRF-VP16ΔMADS down-regulated several of those autophagy and lysosome encoding genes including *Atg9a* (E, Q), *Atg10* (F, R), *Atg7* (J, V) and *Lamp5* (L, X) induced by SOD1 aggregate formation.

In (A-X) N Numbers are indicated by each black dot reflecting one cell culture dish. Statistical testing was performed by one way ANOVA with Tuckey corrections.

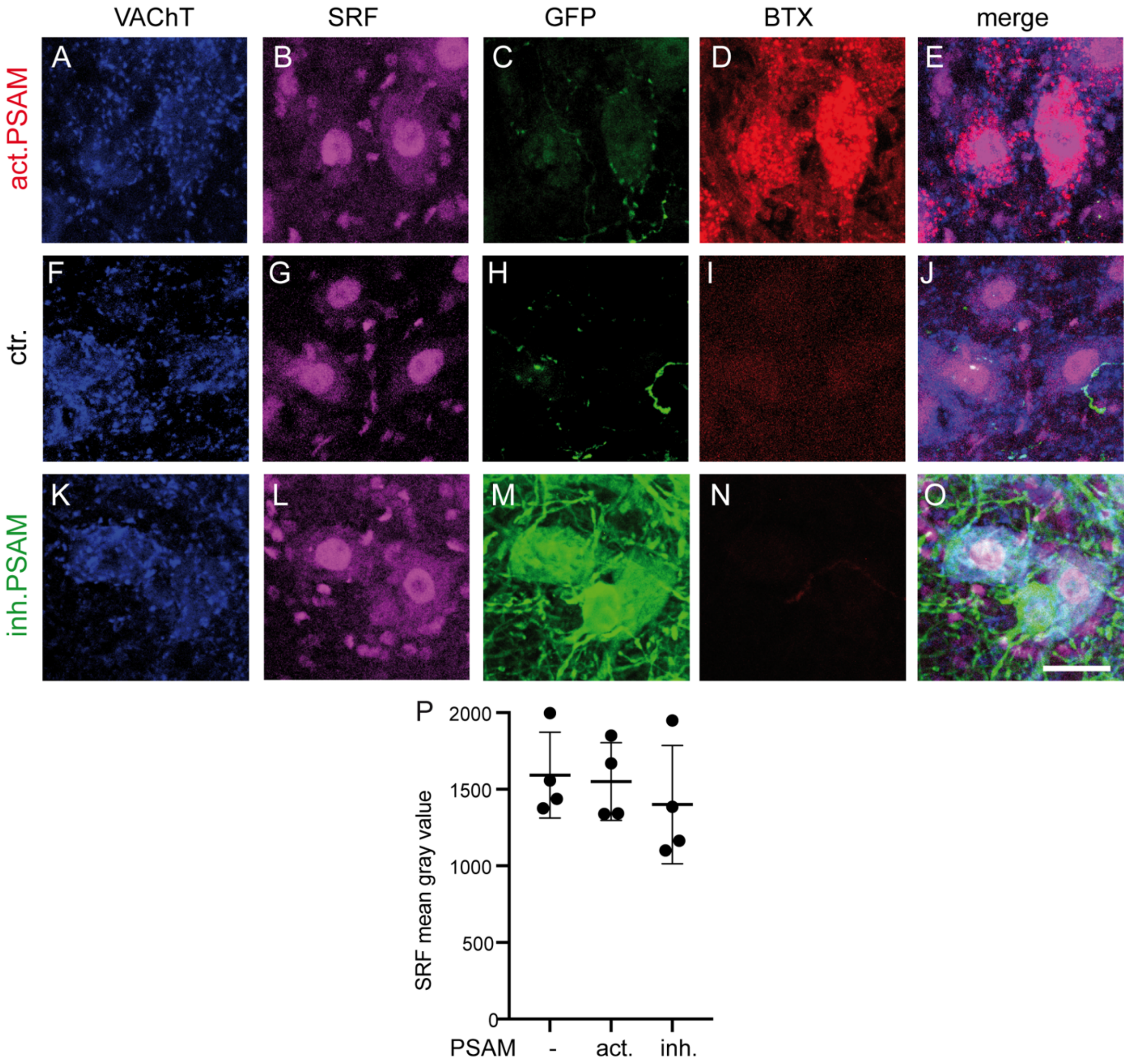

Supp. Fig. 9

SRF expression levels do not change by chemogenetic modulation

MNs of SOD1 mice injected with act. PSAM (A-E), Ctr. (F-J) or inh. PSAM (K-O) virus were stained for the indicated proteins. SRF was restricted to the nucleus of all MNs and SRF expression levels were almost identical between conditions (see quantification in P).

In (P) each dot reflects one mouse analyzed. T-test revealed no statistical differences between conditions. Scale bar (A-O): 30 μm.

Supplementary references

1. Braak, H., Alafuzoff, I., Arzberger, T., Kretzschmar, H., Del Tredici, K. Staging of Alzheimer disease-associated neurofibrillary pathology using paraffin sections and immunocytochemistry. *Acta Neuropathol.* **2006**, 112, 389-404. doi: 10.1007/s00401-006-0127-z.

2. Braak, H., Braak, E. Demonstration of amyloid deposits and neurofibrillary changes in whole brain sections. *Brain Pathol.* **1991**, 1, 213-216. doi: 10.1111/j.1750-3639.1991.tb00661.x.

3. Hyman, B.T., Phelps, C.H., Beach, T.G., Bigio, E.H., Cairns, N.J., Carrillo, M.C., Dickson, D.W., Duyckaerts, C., Frosch, M.P., Masliah, E., Mirra, S.S., Nelson, P.T., Schneider, J.A., Thal, D.R., Thies, B., Trojanowski, J.Q., Vinters, H.V., Montine, T.J. National Institute on Aging-Alzheimer's Association guidelines for the neuropathologic assessment of Alzheimer's disease. *Alzheimers Dement.* **2012**, 8, 1-13. doi: 10.1016/j.jalz.2011.10.007.

4. Braak, H., Del Tredici, K., Rüb, U., de Vos, R.A.I., Jansen Steur, E.N.H., Braak, E. Staging of brain pathology related to sporadic Parkinson's disease. *Neurobiol. Aging* **2003**, 24, 197-211. doi: 10.1016/s0197-4580(02)00065-9.

5. Brettschneider J, Del Tredici K, Toledo JB, Robinson JL, Irwin DJ, Grossman M, Suh E, Van Deerlin VM, Wood EM, Baek Y, Kwong L, Lee EB, Elman L, McCluskey L, Fang L, Feldengut S, Ludolph AC, Lee VM, Braak H, Trojanowski JQ. Stages of pTDP-43 pathology in amyotrophic lateral sclerosis. *Ann. Neurol*. **2013**, 74, 20-38. doi: 10.1002/ana.23937.

6. Brettschneider J, Arai K, Del Tredici K, Toledo JB, Robinson JL, Lee EB, Kuwabara S, Shibuya K, Irwin DJ, Fang L, Van Deerlin VM, Elman L, McCluskey L, Ludolph AC, Lee VM, Braak H, Trojanowski JQ. TDP-43 pathology and neuronal loss in amyotrophic lateral sclerosis spinal cord. *Acta Neuropathol*. **2014**, 128, 423-437. doi: 10.1007/s00401-014-1299-6.

7. Hipp L, Beer J, Kuchler O, Reisser M, Sinske D, Michaelis J, Gebhardt JCM, Knöll B Single-molecule imaging of the transcription factor SRF reveals prolonged chromatin-binding kinetics upon cell stimulation. *Proc Natl Acad Sci U S A*. **2019** Jan 15;116(3):880-889.
